# Female Genital Variation Far Exceeds That of Male Genitalia: A Review of Comparative Anatomy of Clitoris and the Female Lower Reproductive Tract in Theria

**DOI:** 10.1093/icb/icac026

**Published:** 2022-05-07

**Authors:** Mihaela Pavlicev, Anna Nele Herdina, Günter Wagner

**Affiliations:** Department of Evolutionary Biology, University of Vienna, Vienna 1030, Austria; Division of Clinical Virology, Department of Laboratory Medicine, Medical University of Vienna, Vienna 1090, Austria; Department of Ecology and Evolutionary Biology, Yale University, New Haven, CT 06520, USA; Yale Systems Biology Institute, Yale University, West Haven, CT 06516, USA; Department of Obstetrics, Gynecology and Reproductive Sciences, Yale School of Medicine, New Haven, CT 06510, USA

## Abstract

A review of the literature on the anatomy of the lower female genital tract in therian mammals reveals, contrary to the general perception, a large amount of inter-specific variation. Variation in females is anatomically more radical than that in the male genitalia. It includes the absence of whole anatomical units, like the cervix in many Xenarthra, or the absence of the urogenital sinus (UGS), as well as the complete spatial separation of the external clitoral parts from the genital canal (either vagina or UGS). A preliminary phylogenetic analysis shows two patterns. Some morphs are unique to early branching clades, like the absence of the cervix, while others arose multiple times independently, like the flattening out or loss of the UGS, or the extreme elongation of the clitoris. Based on available information, the ancestral eutherian configuration of the external female genitalia included a cervix, a single vaginal segment, a tubular UGS, and an unperforated clitoris close to the entrance of the genital canal. The evidence for either bilobed or unitary glandes clitorides is ambivalent. Despite the wealth of information available, many gaps in knowledge remain and will require a community-wide effort to come to a more robust model of female genital evolutionary patterns.

## Introduction

The phallus is a shared derived characteristic of amniotes and is thus of a single evolutionary origin (Sanger et al. 2015). It is present in both sexes, the penis in males and the clitoris in females. Comparative anatomy, physiology, and function of the male phallus are well-studied (e.g., Dixson and Anderson 2001; Dixson 2012; Gredler et al. 2014; Gredler 2016; Schutz et al. 2016), whereas similarly detailed descriptions of the clitoris are scarce, in particular when seeking broad comparative accounts. Multiple reasons explain this situation. One practical reason is that female genitalia are often less conspicuous than male genitalia. However, the relatively small external parts are paired with internal parts, which in size match their male counterparts. Internal parts are less well-investigated in both sexes, as they are intricately embedded in the connective tissue and the musculature of the pelvic floor and thus difficult to dissect. Another practical reason may be that females are considered more valuable than males in breeding, presenting another obstacle to invasive anatomical study. Finally, the physiological variation introduced by female ovarian cycling likely made female animals less preferred research subjects in general. Other reasons for the current scarcity of information are more theoretical, such as the conclusion that female genitalia are allegedly uniform (thus requiring little explanation) because sexual selection acts on males, or the assumption that the clitoris lacks biological function. Additional difficulty is that a large part of the relevant anatomical literature was published in German (and perhaps other languages), such that a large knowledge base is difficult to access for many researchers. As these and other reasons for the lack of attention to clitoral anatomy are being increasingly overcome in recent decades, we observe intensified research into the details of the female reproductive anatomy, its function and evolution (Puppo 2013; Cunha et al. 2014; Lima et al. 2015; Pavlicev and Wagner 2016; Sinclair et al. 2017; Orbach et al. 2019; Pavlicev et al. 2019; Levin 2019; Orbach et al. 2021; Brennan et al. 2022). To further foster this direction of research, we aim at summarizing the comparative anatomical information currently available.

In the present contribution, we review the available literature on the anatomy of the female phallus, i.e., the clitoris, and place this knowledge into an anatomical and phylogenetic framework. Such comparative compilation is crucial for two reasons. First, the lack of comparative studies is the likely cause of inconsistencies in the terminology and anatomical interpretations further impeding scientific work and leading to poor understanding of female genital evolution and its causes. Second, comparative genital anatomy, when combined with other characteristics of mammalian reproduction such as ovarian cycle characteristics and social structure, forms the basis for understanding female genital function and sexuality, as well as their evolution. Our own interest in this area was sparked by the apparent correlation between the nature of the ovarian cycle and genital anatomy, in particular the type of ovulation and the presence of the urogenital sinus (UGS: Pavlicev and Wagner 2016; Pavlicev et al. 2019).

Expanded comparative data on reproductive anatomy, when combined with species-specific reproductive physiology, will enable us to test hypotheses about female sexual function and evolution, based on the idea that evolutionary changes in sexual function will be reflected in changes in genital anatomy. A solid understanding of the anatomical evolution of female genitalia is thus a necessary condition for understanding the evolution of female sexual function and behavior.

## Evolution of the phallus

The phallus originated in the stem lineage of land vertebrates, the amniotes: birds, reptiles, and mammals, despite its absence in several amniote lineages (e.g., songbirds). Classical anatomical literature (e.g., Gegenbaur 1898), as well as more recent studies, note the presence of a genital anlage in the ventral wall of the cloaca in early developmental stages in species that lack a phallus in the later stages of development, including the early branching lineages such as *Sphenodon*, aka Tuatara, a sister species of squamates (Sanger et al. 2015). This suggests a common origin with subsequent losses in adults of multiple amniote lineages, rather than multiple independent origins. While the amniote phallus is associated with internal fertilization, the latter does evolve several times independently outside of amniotes in even earlier branching lineages, such as sharks, some teleosts, and amphibians. However, the functionally similar male structures used for internal fertilization in these lineages have different anatomical bases (e.g., shark claspers are modified from pelvic fins: Gerhardt 1939; the gonopodium, intromittent organ of *Xiphophoruous* fish, is a modified anal fin: Langer 1913), and will not be further addressed here.

The evolutionary origin of the mammalian phallus thus coincides with the origin of internal fertilization in stem amniotes, predating the diversification of crown amniotes. It is unclear, which exact factors have been crucial for the *origin* of direct sperm deposition into the female cloaca (e.g., male–male competition). The *consequence* of internal fertilization, however, is clearer: it emancipated amniote fertilization from the aquatic environment. The directionality of gamete deposition is overwhelmingly uniform—that is, in most species, the males deposit gametes into the female reproductive tract, where fertilization occurs (rather than the other way around as in seahorses). It, therefore, appears likely that the evolutionary origin and the early evolution of the phallus have been driven by its male reproductive function, although homologous structures are present in both sexes. The clitoral functions have thus likely arisen secondarily. Another interesting aspect is that in amniotes the intromittent organ arose as a Type I novelty (Wagner 2014), i.e., a novel body part, while in other lineages intromittent organs arise as a modification of existing body parts (Type II novelty), e.g., pelvic or anal fins. In these latter cases, there is little if any modification of the female counterpart, while in amniotes the phallus is also a part of the female anatomy.

## Development of mammalian phallus

We base the general outline of embryonal development on descriptions of humans and mice, as comparative information at these early stages is rare—however, some degree of deviation must be expected. The embryonic origin of the phallus, its general structure and histology (including innervation, vascularization, and muscularization), as well as its anatomical position are clearly homologous between male and female phalli. The two structures are indistinguishable during their early development, diverging relatively late in development (Yamada et al. 2006; Carlson 2014; Cunha et al. 2018, 2020)

**Fig. 1:**
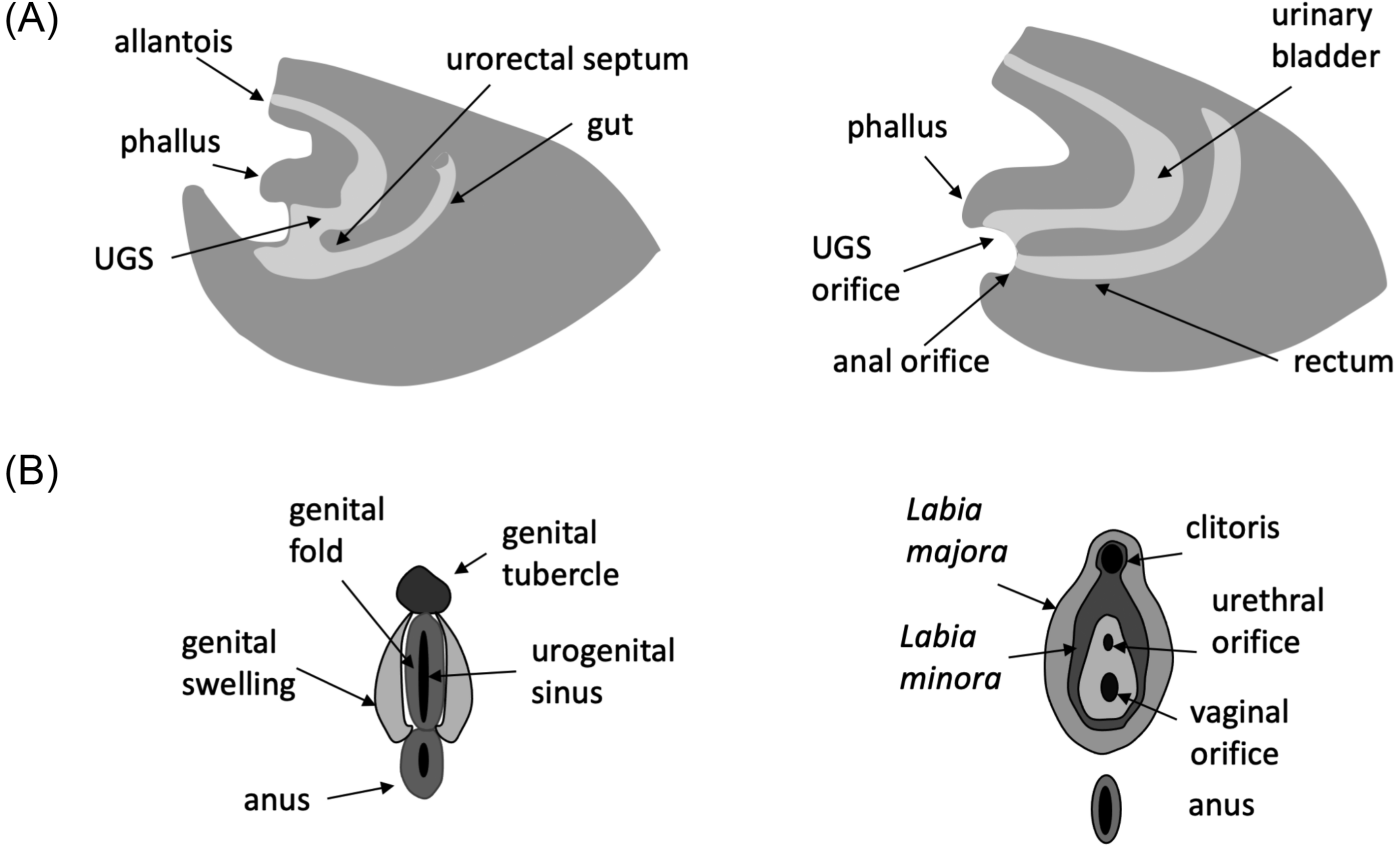
Genital development (human). (**A**) During early development, the gut is separated from what becomes a UGS by the growing urorectal septum, and subsequently the two separate orifices form. Phallus will develop ventrally from UGS. (**B**) External genitalia formation at the undifferentiated stage on the left and differentiated (female) stage on the right. Figures inspired by Carlson (2014).

At the beginning of phallus development, the urogenital system consists of a cloaca, connecting the endodermal extraembryonic allantoic sac and the gut (Fig. 1A). The mesodermal urinary and genital ducts (Wolffian duct in male and Müllerian in female) open into the cloaca, whereas the cloacal membrane separating the cloaca from the ectodermal proctodaeum is still intact, and thus the external ano-genital openings are lacking. The beginning of phallus development is marked by the genital swellings arising at the ventral rim of the cloaca, the site of the future external genitalia. These form the paired genital folds, which later often fuse at the midline to form a single genital tubercle (but remain paired in reptile hemipenes/hemiclitorides, or partially fused as for instance in the distally split phallus of opossum). At the same time the gut separates dorsally from the rest of the cloaca by the development of urorectal septum (Fig. 1A), forming the rectum with a distinct orifice (anus), whereas the remaining part of the cloaca is now called the UGS, and opens in an orifice arising between the genital folds (Fig. 1B). The genital tubercle develops into the phallus, which up to this stage is morphologically sex-invariant (note that sex differentiation at the level of cell types starts earlier; Armfield and Cohn 2021). From this developmental stage on, the development of the male and female external genitalia diverges visibly under the influence of gonadal hormones.

The subsequent genital development involves the integration of the external genitalia with the inherently sexually dimorphic urogenital systems. In male therian mammals, this means the integration of the genital and urinary duct into the phallus. This duct is composed of direct derivatives of the mesodermal ducts (mesonephros; Wolffian duct), which give rise to genital tubes (*vasa deferentia*) connecting testes to the urethra. The resulting single duct, urethra, thus corresponds topologically to the UGS, and carries both urine and sperm. This pattern is conserved in male eutherians—the penis invariably envelops a single duct with joint function. Thus, despite the wide diversity of the late-developing penile characteristics (presence/absence of the penile bone, spines, overall size, the size and number of erectile bodies, skin folds, and so on), the integration between UGS (confluence of genital and urinary tracts) and penis appears unchanged across Eutheria, as reported in rich comparative compendium by Gerhardt (1939).

The development of the female external genitalia is considerably more variable across species. In females the Wolffian ducts degrade, with the exception of some minor vestigial structures (e.g., Gartner's ducts). The female genital tract connecting the ovaries with an external orifice instead develops from the paired mesodermal Müllerian ducts (paramesonephric duct; an anatomical part which degrades in males). This anatomical entity eventually differentiates into oviduct, uterus, cervix and potentially the proximal portion of the vagina. The developmental origin and evolutionary individuality of the vagina (as identified by the typical squamous epithelium) are still disputed, even for humans. A commonly held model for human development (e.g., Carlson 2014) is that the upper part of the vagina develops from fused parts of the Müllerian ducts, and a solid tissue block from the Müllerian tubercle (so-called vaginal plate, a proliferation of endoderm of UGS at the location of contact with mesonephros) hollows out to form the complete lower part. Ulfelder and Robboy (1976), studying congenital defects of the vagina, have suggested contributions of Müllerian ducts as well as UGS, where the epithelial cells from UGS migrate into the part formed by the Müllerian ducts, thus both contributing to vagina [similar proposal was made by Bulmer (1957)]. Recent work by Robboy et al. (2017) and Cunha and colleagues (summarized in Cunha et al. 2018), using protein markers to trace epithelia in vaginal development shows that UGS-reactive FOXA1 expression eventually spreads up to the cervix, thereby transforming the simple into stratified squamous epithelium—consistent with the earlier proposal. Starck (1975), drawing on his work on mice, rats, hamsters, rabbits, dogs, cows, and humans, suggests variation in vaginal development across species, which could indicate that the vagina's identity might be formed by different cell lineages in different species and is thus not linked to the embryonic origin of the cells but likely by the activation of a shared vaginal gene regulatory network.

The UGS, i.e., the confluence of urethra and the derivative of the Müllerian ducts, develops as the urethra, coming from the bladder, opens into the reproductive tract. However, the persistence of the UGS beyond embryonic stages, and its spatial relation to the clitoris are variable (see below).

## Major parts and terminology

Given the large amount of interspecific variation in the anatomy of the female external genitalia in therian mammals, it is difficult to establish a terminology that can be applied to all species and anatomical configurations. In addition, the amount and detail of information available for different species is quite variable, with developmental information limited to a small handful of species. Thus, in many cases the meaning of the anatomical terms identified in the literature remains unclear. Furthermore, embryological units do not always correspond to adult morphological structures. As noted above, an example is the human vagina, to which two different embryological and genetic units are likely contributing, the distal (lower) and the proximal (upper) vagina. Keeping these limitations in mind, we propose a terminology that can be applied to most anatomical descriptions available in the literature.

**Fig. 2:**
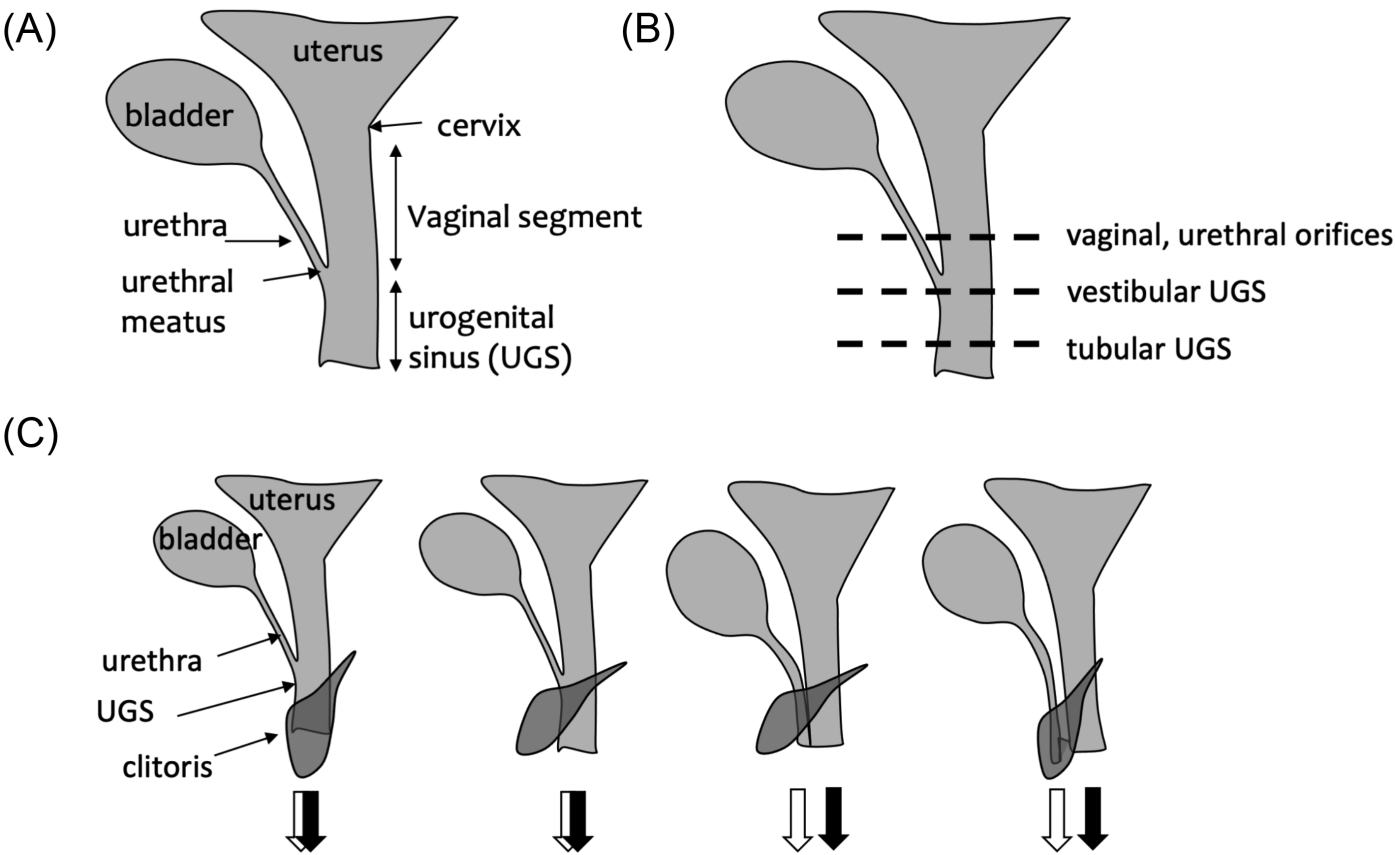
Dimensions of female therian genital variation. (**A**) Scheme showing the naming of parts used in the present paper. (**B**) The variation in the number and nature of orifices can be classified by the relative position of the urethra joining the reproductive tract. (**C**) The variation in the relation of clitoral apparatus to the urethral and vaginal openings. The two patterns on the left show clitoris associated with UGS; either being traversed by the UGS, or separate. The two patterns on the right show cases in which urethra opens in separate orifice from the vaginal segment, and the clitoris is either ventral to, or traversed by the urethra.

There are three anatomical landmarks that can be used to conceptually regionalize the female lower genital tract: the cervix, the urethral opening (meatus), and the *Corpus clitoridis*. Most anteriorly, there is the transition from the uterus to the genitals, which is usually marked by the cervix, a quite distinct structure in most therian mammals. In Xenarthra (anteaters, sloths, and armadillos) and in the afrotherian elephant shrews, the utero-vaginal canal, which topologically corresponds to the cervix and the vagina of other mammals, has a different structure (Cetica et al. 2005), as will be described below. The urethra, guiding urine from the bladder, can open either into a genital canal or directly to the outside. If it opens into a genital canal, we call the segment between the cervix and the urethral meatus the *vaginal segment* (Fig. 2A). Note that this term indicates a topological unit and not necessarily part of, or all of, the copulatory canal. The vagina proper can be shorter than copulatory canal if the urethral meatus opens at a distance from the genital openin (Fig. 2B). Then there is the *Corpus clitoridis*, which can be located inside the genital canal, at its rim, or outside of it (Fig. 2C).

If the genital tract continues after the urethral meatus, this segment of the genital canal will be called UGS, in accordance with much of the comparative anatomical and embryological literature. If, however, the genital tract widens, so that what topologically corresponds to the UGS and even part of the vaginal segment is not a canal but flattened out, then this part is called the *vaginal vestibule*. Note that a tubular, longer UGS can also broaden at its caudal end, and is often described in the literature as vestibulum—we treat, therefore, information about the presence of vestibulum independently from the information on the presence of UGS. For instance, in humans the vagina is shorter than the vaginal segment as defined above (because the urethral meatus opens into the vestibule) and the vestibule includes part of the vaginal segment and the UGS (Fig. 2A). We limit the term *vulva* for the part of the pudendum that lies outside the *Labia minora*.

Further, we use a meta-term *genital canal* for any tube-like structure, regardless of whether it is derived from the UGS, the vaginal segment or both, and regardless of whether it is engaged during copulation. For instance, in some animals (e.g., elephants) the genital canal is longer than the penis. The genital canal is sometimes called the “vagina,” from a functional point of view, but such a term would hinder the evolutionary comparative work, as relative contributions to a genital canal of developmentally different parts differ across species.

Phallus is furthermore associated with two skin folds, preputium (prepuce) and frenulum. Both are parts of *Labia minora*. Prepuce is also referred to as clitoral hood as it is described in many species to cover the *Corpus or Glans clitoridis*.

**Fig. 3:**
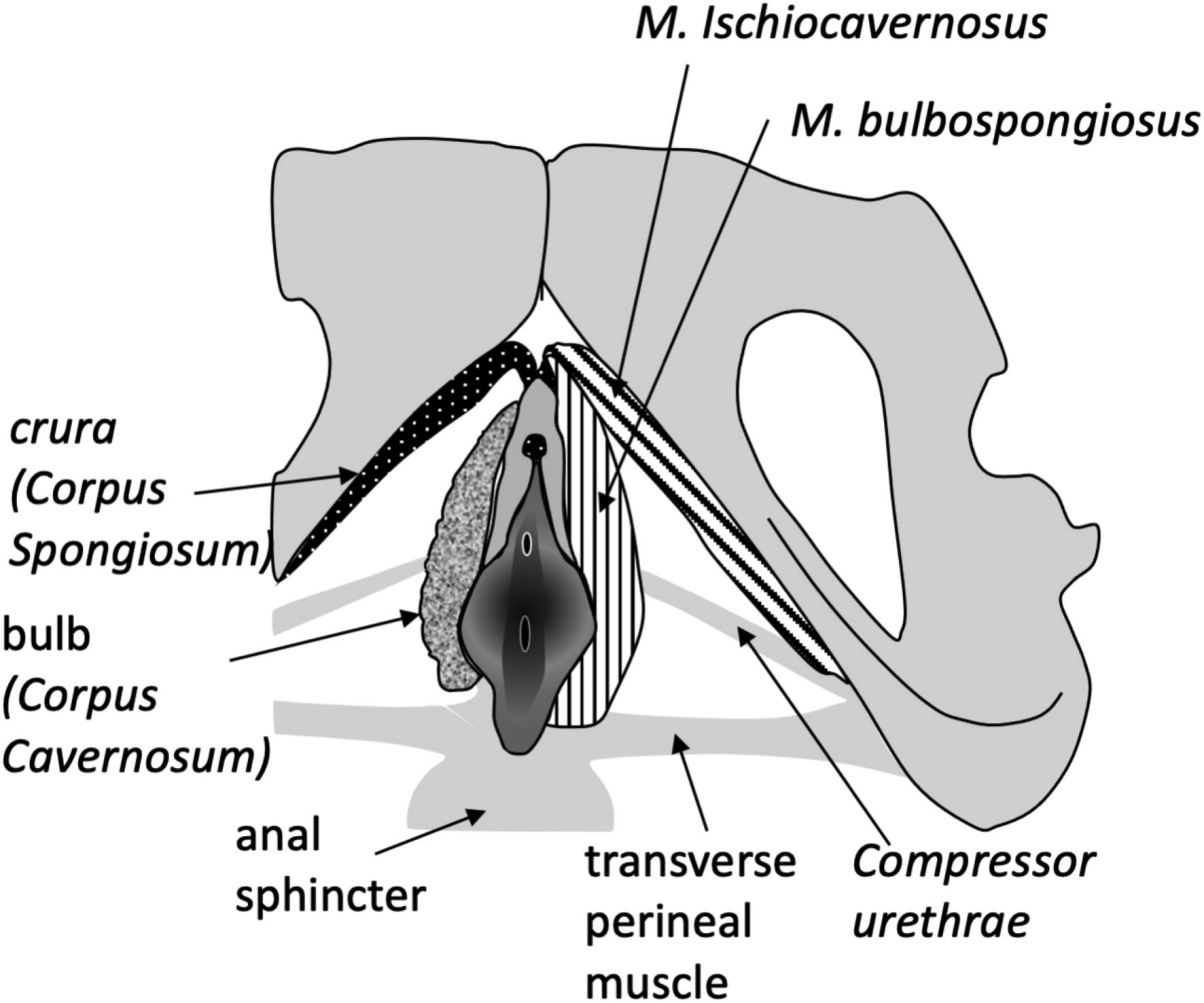
Overall position of the clitoral apparatus, and its attachment to the pelvis and parts of pelvic floor. The human structure is shown

Finally, we need to distinguish two types of erectile tissues in the phallus, the *Corpus cavernosum* (CC) and the *Corpus spongiosum* (CS) (Fig. 3). The first is the erectile tissue consisting of a meshwork of smooth muscle cells forming a blood sinus responsible for stiffness during erection. In contrast, the CS is distinct from the CC and the surrounding tissue due to a high level of elastic fibers (van Turnhout et al. 1995), in addition to collagen and smooth muscle fibers. For example, a quantitative study of the rabbit penis found the CS to have a volumetric concentration of elastic fibers of 32% compared to 25% in CC. During erection, the CS therefore remains elastic. The shape and contribution of these two tissues to the female phallus is variable among species (see below).

## Dimensions of genital variation

In contrast to the penis, the mammalian clitoris exhibits a wide range of structural and positional variation. Before delving into the systematic survey, we provide general phenotypic dimensions, along which female genitalia differ. These dimensions often do not represent overall evolutionary trends but rather are found repeatedly in various clades.

Female external genitalia and their integration with the lower urogenital tract vary in multiple dimensions. One dimension is the relation between the genital and urinary tract. Ancestral adult mammalian anatomy, present in Monotremes and to some extent in Marsupials (Marsupial moles), involves the cloaca, the cavity into which genital and urinary tracts as well as rectum empty. In adult eutherians and at least some marsupials, the rectum is separated from the urogenital ducts. Variation persists in whether urinary and genital ducts also exit the body separately, or share some extent of the tract, forming the UGS with a single orifice (similar to male). Where along the genital tract the confluence of the urinary and genital tract occurs is also highly variable, ranging from the very proximal part close to the utero-vaginal canal (which replaces the cervix) in Xenarthra, or very close to the end of the genital canal, or in a flat vaginal vestibulum (e.g., humans). If the vaginal segment is considered as the proximal part of the genital canal extending from cervix to the urethral meatus, and the UGS as the distal part extending caudally of the urethral meatus, the relative length of UGS varies from a very long to a very short segment (vestibulum), or even complete absence. Together, the vaginal segment and (if present) the UGS, form the genital canal, if both are tube-shaped (Fig. 2).

Further variability concerns the relation of the clitoris to the urethra and the UGS (Fig. 2B). Clitoral *Corpora cavernosa* and the *Corpus clitoridis* are located ventrally along the urethra. The external *Corpus clitoridis* emerges either in the lumen of the UGS, the vestibulum, or on the rim of the vestibulum. As mentioned above, in many species (e.g., many rodents), vagina and urethra open as separate orifices (no UGS), and parts of the *Corpus* and *Glans clitoridis* in this case are exposed on external body surface, ventrally to the urethral orifice. In a few mammalian species, spread among different clades, the *Corpus clitoridis* is traversed by either the urethra or the UGS and assumes a penis-like morphology. This often coincides with a hypertrophied clitoris, but clitoral elongation, up to the length of male phallus, also occurs in various further clades without embedding any of the ducts. As explained below, these elongated clitorides differ widely in structure, as well as in developmental origin.

Despite clear sexual dimorphism, some aspects of the clitoris covary with penile characters across species. Variation in the presence of the *Os clitoridis* (baubellum or clitoral bone) to some extent follows variation of the *Os penis*, the baculum (Lough-Stevens et al. 2018; Spani et al. 2021). Both phalli also appear to covary in the extent of bifurcation.

Less well-documented is the variation in the presence and size of erectile bodies, in particular their internal parts. In general, the joined *Corpora cavernosa*, which build the main portion of a mostly single-bodied external phallus, continue internally as separated paired roots, called *Crurae*, extending laterally along pubic bones toward the ischium (Fig. 3). They provide attachment to the paired *Musculus ischiocavernosus*, which attaches the phallus to the bony pelvis. The second paired erectile bodies, the *Corpora spongiosa* (or sometimes, confusingly, called *Corpora cavernosum urethrae*), described in several species including human, form paired internal bulbs at the basis of the phallus, extend along the phallic corpus and build the *glans* at the tip of the phallus. This tissue is often associated with the urethra/UGS and in males provides a single column embedding the UGS (i.e., the distal urethra) in penis and ensuring that the UGS remains open for ejaculation during erection. Other ligaments and muscles of the perineum are associated with the male as well as the female phalli and will be addressed below only where sufficient information has been found.

Overall, it is noteworthy that the clitoral parts have often been modified within single lineages and over short evolutionary times. This is suggested by the phylogenetic distribution and by the distinct anatomy involved in generating superficially similar phenotypes. A good example is the evolution of the particularly hypertrophied genitalia. These well-known large clitoral parts arise in some species of insectivores (moles), bats (some Noctilionoidea; McCarthy et al. 1988, Carter and Mess 2008), carnivores (spotted hyena; Cunha et al. 2014), and primates (lemurs and NWMs; Drea and Weil 2008). However, they are anatomically distinct: while the enlarged clitoris of hyena encloses the UGS, the enlarged clitoris in lemur only encloses the urethra, whereas the vagina opens separately at the clitoral basis and hence lacking a UGS altogether. The long clitoral structure of the Spider monkey (genus Ateles, the NWMs) on the other hand, encloses neither the genital nor the urethral canal. Finally, the enlarged perineal structure of moles is not even clitoris proper, but rather the prepuce, i.e., the skin fold enclosing the *Glans clitoridis*. The morphology of elongated clitorides found in some species of *Lophostoma* bats has yet to be described.

## Systematic overview of therian clitoral anatomy and position

This section reviews the available information on clitoral anatomy, with the phylogenetic context in mind. A brief formal phylogenetic analysis will be presented after this section. Neighboring tissues (vagina, UGS, and perineal musculature) will be addressed along with the clitoris, whenever available information permits.

### Marsupials

The general anatomy of the marsupial clitoris appears to follow that of the penis: in the Virginia opossum (*Didelphis virginiana*; McCrady 1938) as well as in Tasmanian devil (*Sarcophilus satanicus*; Flynn 1910, 1911), the *Corpus clitoridis* is described as bifurcated, protruding in the UGS at its posterior end (opossum). *Corpus cavernosum* is described to fill the external part of the clitoris, and no information is available about the *Crurae*.

In the *Monodelphis* and *Didelphis* opossums, two uteri with distinct cervices and three vaginae are described. The UGS is separate from, but opens close to, the anus (McCrady 1938 and pers. observation). Together, both openings are surrounded by a shallow skin fold creating the superficial appearance of a cloaca. In the Tasmanian devil, a distinct uterine “neck” is described, but from the description it is not clear whether the author means a single cervix, which would mean that the uterus is divided into two uterine horns, rather than the two uteri. A total of two lateral vaginae are mentioned with a median vaginal apparatus (i.e., three vaginae), but a single, very long UGS, which opens into the cloaca (Flynn 1911). Re-examination of this species would be highly desirable.

### Xenarthra

In this most basally branching eutherian group, the information on genital anatomy is available for Brown-throated sloth (*Bradypus variegatus*, Favoretto et al. 2016), Lesser anteater, (*Tamandua tetradactyla*, Hayssen 2011; Rossi et al. 2011), and several species of armadillo (Cetica et al. 2005; Pocock 1924b). In many xenarthrans, the urethra joins the reproductive tract immediately caudal to the so-called “uterovaginal canal,” also sometimes referred to as cervix. Both names of this narrow structure are misleading, as it relates uterus with UGS rather than with vagina, and it structurally differs from the cervix of other mammals. It is lined by the columnar epithelium, similar to the uterus or the endocervix in most other eutherian lineages, yet the distinct glands, similar to cervical glands are described. Hence, a distinct cervix, as well as a true vagina (the latter characterized by the squamous stratified epithelium), are missing, and the reproductive tract continues posteriorly as the UGS. An exception appears to be the Southern three-banded armadillo (*Tolypeutes matacus)*, for which a true vagina has been reported (Cetica et al. 2005). A double vagina separated by a sagittal septum has been described in *Bradypus* by Giersberg and Rietschel (1968). It is noteworthy that the vagina is often described based on the gross-morphological characters (relative position and form), such as in the nine-banded armadillo (*Dasypus novemcinctus*; Enders and Buchanan 1959). In the latter case, its detailed morphological characterization (e.g., the presence of columnar epithelium) suggests homology with the uterovaginal canal as described in other xenarthrans (also suggested for *Dasypus* by Giersberg and Rietschel 1968). This aspect of xenarthran anatomy in particular requires further attention.

The xenarthran *Corpus clitoridis* is very variable and its bipartite structure can still be observed in this group. Brown-throated sloth (*Bradypus variegatus*) presents a bipartite (bilobed) clitoris with paired *Crurae*, bodies, and glandes (Favoretto et al. 2016). The paired *Corpora cavernosa* in the clitoral body remain separated by a septum. In contrast, the pale-throated sloth (*Bradypus tridactylus*) was described to have a simple small un-grooved clitoris on the vulvar edge (Pocock 1924b). Pocock (1924b) also described the external genitalia of several species of the two armadillo families, Dasypodidae and Chlamyphoridae, and three species of anteaters. The author noted that the external clitoris of the Nine-banded Armadillo (*D. novemcinctus*, fam. Dasypodidae, the long-nosed armadillos) is hidden by large labia, often confused with a male penis. Also, the visible clitoris of *Chlamyphorus* (fam. Chlamyphoridae) is small. In contrast, the clitorides of Chlamyphoridae genera *Euphractus, Tolypeutes*, and *Zaedyus* are elongated, penis-like singular structures. However, the similarity to a penis does not extend to internal structures: in all cases, the urogenital meatus is located dorsally to the *Corpus clitoridis* at its basis, and the external *Corpus clitoridis*is often grooved on its dorsal side.

Anteaters appear intermediate in their clitoral anatomy between sloths with stronger bipartite characteristics and armadillos with simpler, unified structures. Pocock (1924b) describes anteater species of two genera, Tamandua and Cyclopes. In Lesser Anteater (*T. tetradactyla*), the clitoris is simple and undivided, covered by hairy labia (Hayssen 2011; Pocock 1924b). In Silky Anteater (*Cyclopes didactylus)*, the clitoris is similar to that of the Lesser Anteater, yet bilobed. Uterovaginal canal in Lesser Anteater is lined by simple columnar mucous epithelium, which turns into transitional epithelium of the UGS after confluence with urethra, thus lacking a vaginal segment (Rossi et al. 2011). The UGS opens in the vestibular cleft (vulva).

### Afrotheria

In Afrotheria, the genital anatomy is described for the African (*Loxodonta africana*) and Asian elephants (*Elephas maximus*; Perry 1951; Balke et al. 1988), Hyrax (Procaviidae; Lonsky 1903), multiple species of elephant shrews (genus *Elephantulus*; Van der Horst 1942; Tripp 1971), Aardvark (*Orycteropus afer*; Pocock 1924a), and Amazonian manatee (*Trichechus inunguis;*Rodrigues et al. 2008). Both elephant species are known for their very long female reproductive tract, passing through the pelvis, but then curving ventrally, with the urogenital orifice at a far distance from the anus, directed downwards rather than backwards. Some discrepancies exist in description of the cervix, vagina, and UGS as well as the hymen. This review follows Perry's (1951) account of these differences and describes the common structures of African and Asian elephants. Vagina is present, followed by a narrow transition (covered by the hymen) into a very long UGS representing approximately 50% of the length of the total genital tract (2-2.5 m long). UGS is composed of the intrapelvic and extrapelvic portions, of which the latter is reported to correspond to the full length of the very long clitoris. *The Corpora cavernosa* extend along the UGS about half of its length (i.e., approximately one-fourth of the reproductive tract). *The Ischiocavernosus* muscle [called “*retractor clitoridis*” by Perry (1951)] inserts to a tendon close to the *Glans*. The *Corpus clitoridis* reaches into the ventral edge of vulva, the imperforate glans visible. The clitoral body, formed by the fusion of the *Crurae*, is reported to line the ventral wall of the UGS and be of 18 cm (7 inches) in length and 1.25 cm in diameter, the last 10 cm being pendulous. It is not clear where the clitoral shaft protrudes into the UGS—but given the size of the *Corpus clitoridis*, it would mean that this occurs about 40 cm cranial from the vestibulum, in the UGS. Bulbs are not described.

Hyrax female genital anatomy includes a vagina, which is joined by the urethra 3 cm caudal to the cervix (Lonsky 1903). UGS is thus present and broadens distally to form a *Vestibulum vaginae*—which refers here to the wide caudal end of the UGS. The clitoris protrudes on the ventral side of vestibulum with button-like grooved glans and distinct preputium and frenulum. Clitoral corpus is partially unattached. Lonsky mentions *Crurae* or bulbs only in males, there is also no mention of a baubellum (Lonsky 1903).

In Amazonian manatee, the vagina as well as UGS are described. The external clitoris protrudes into the UGS just before the opening of the UGS to vulva and is visible. The length of the free clitoral parts is described to be 28 mm in adults (Rodrigues et al. 2008). In Aardvark (*O. afer*), only the description of external parts is available. Here, the clitoris is described as bilobed and long, extending beyond the genital orifice (Pocock 1924a).

Elephant shrews (Tupaiidae) manifest strikingly different reproductive anatomy than other Afrotheria in that they, as also seen in Xenarthra, lack a cervix and true vagina. This was first noted by Van der Horst (1942) in Eastern Rock elephant shrew (*Elephantulus myurus*) and confirmed for six further species of elephant shrews (*E. fuscipes, rufescens, rozeti, intufi, brachyrhynchus*, and *edwardii*) by Tripp (1971). Cervix and vaginal segment in the reproductive tract of elephant shrews are topologically replaced by the elongation of uterus, akin to the uterovaginal canal described in Xenarthra. No description of lower genital tract and genitalia was found for these species.

### Laurasiatheria

In laurasiatherians, information is available for Eulipotyphla, Chiroptera, Carnivora, and Ungulata including Cetacea (Meek 1918; Rommel et al. 2007).

Old and New world moles are a well-studied group of Eulipotyphla, raising particular interest due to species with notable genitalia in both sexes and due to the presence of ovotestis, the inclusion of highly testosterone-producing tissue in the ovary (Rubenstein et al. 2003; Sinclair et al. 2016, 2017). Enlarged female genitalia had been thought to be caused by the production of testosterone during fetal development (Whitworth et al. 1999), however, it has been shown recently that this enlarged structure is in fact the prepuce housing a much smaller clitoris (of the size of the male penis), rather than being an enlarged phallus itself. In congruence, some New World mole species (e.g., genera *Scalopus* and *Scapanus*) develop enlarged prepuce in absence of ovotestis (Rubenstein et al. 2003). The mole clitoris is thus similar in structure to mouse clitoris in that it is an internal cylindrical structure located along the urethra and defined on the ventral side by a U-shaped epithelial lamina (i.e., not defining the fully mobile separate cylinder such as is the case for penis). The urethra runs on the dorsal side of this semi-defined structure, while the prepuce envelops both.

Mole species vary in the degree to which the clitoris is attached to the adjacent tissue, in the size of the prepuce and its extension beyond the body, as well as in the presence of an *Os clitoridis* (Sinclair et al. 2017). The vaginal opening is mostly separate, however, in some species the distal part of the urethra joins the vagina which runs just parallel to it on the dorsal side. The clitoris is characterized by a CC but lacks a CS.

Bats (Chiroptera) are the mammalian clade with the second greatest number of species, after Rodentia, and their reproduction is well-studied. Anatomical descriptions of female reproductive tract often focus on the upper parts of the reproductive tract (ovaries and uterus) and are available in many species, distributed across the two groups of Yango- and Yinpterochiroptera (Hood and Smith 1983). Female genitalia have, however, received less attention. Hood (1989) summarized the reproductive tract of Macroglossusinae (Macroglossinae), a subfamily of Yinpterochiroptera (Pteripodidae), and compared them to several Yangochiropteran families. As other authors, he points out great variability of uterus type and cervix length and shape among bats (Hood 1989; Hood and Smith 1983; Carter et al. 2008). Among Yangochiroptera, uterine type includes everything from the bicornuate uterus, such as in Myzopodidae, the Madagascar sucker-footed bats (Carter et al. 2008), and uterus simplex, such as in *Artibeus lituratus*, the great fruit-eating bat of the family Phyllostomidae (Rodrigues et al. 2019). Within Yinpterochiroptera the common uterine body (as opposed to horns) can be very short (*Megaloglossus sp*.) to substantial (*Macroglossus sp*.; Hood 1989). Cervix length represents one-third of the uterus in Yinpterochiropteran families (megadermatids, hipposiderids, or rhinopomatids) and Yangochiropteran families (emballonurids or phyllostomoids), whereas it is half of the uterus in other Yangochiropteran families (vespertilionids and mystacinids). The cervix is traversed by a single canal in most families of bats; however, two cervical canals occur in single species of both Yinpterochiroptera (pteropodids) as well as Yangochiroptera (emballonurid family). There is thus a huge variation in the extent of fusion of Müllerian duct-derivatives during the development in this group. A vagina is present and covered by a multilayered squamous epithelium (Rodrigues et al. 2019). In some species of vespertilionids (Molossidea) and emballonurids (both Yangochiroptera) the urethral meatus is described to open into the vestibulum separately from the vagina. Thus, no tubular UGS is present (Crichton and Kruztsch 1987; Matthews 1942). The vulvar opening in Yinpterochiroptera and several families of Yangochiroptera is a transverse slit ventral to the anus and covered with an externally placed, flattened clitoris (Hood 1989). Matthews (1942) describes *Corpora cavernosa clitoridis* of *Coleura afra* (emballonurid bat), and Crichton and Kruztsch (1987) describe paired *Corpora cavernosa clitoridis* enclosing another erectile space containing a slender sigmoid *Os clitoridis* or baubellum in *Ozimops* (*Mormopterus*) and*planiceps* (vespertilionid bat). In some chiropteran species, a baubellum is present (Lough-Stevens et al. 2018). The clitoris is reportedly small for Yangochiropteran families Mezopodidae, Vespertilinoidea and Emballonuroidea, but certain species show an elongated clitoris, such as members of Noctilinoidea (Carter et al. 2008) and Mormoopidea (e.g.,*Pteronotus macleayii;*Mancina 2005).

In the Cetaceans, the vagina and urethra open into the vestibulum separately, no tubular UGS is observed. The vestibulum lies in the slit to which also anus and mammary slits open. In most species examined, the external clitoral parts (corpus and glans) protrude into the vestibulum close to the urethral meatus, just after the narrow transition between vagina and vestibulum. The clitoris is generally well-developed with prepuce and prominent glans and can be seen reaching beyond the vestibular slit. It consists of a well-developed and erectile CC (Meek 1918; Morejohn and Baltz 1972; Rommel et al. 2007). Meek (1918) investigated Harbour porpoise (*Phoecena phoecena*) and Greenland whale (*Balaena mysticetus*). In porpoise, Meek noted that paired *Corpora cavernosa* fuse before entering clitoral shaft. Distally, they run along the so-called *Erector clitoridis* ( = the *M. ischiocavernosus*) and attach to the pelvic bone. Meek reported that clitoral corpus as well as glans in porpoise are invaded by CC (possibly spongiosum?). Paired *Crurae*, which are attached by *M. ischiocavernosus* to the pelvic bone, however, are reported to not consist of erectile tissue [Meek 1918; but see Brennan et al. (2022)]. A histological investigation of the Bottlenose dolphin (*Tursiops truncatus*) clitoris showed a high nerve density and dense nerve-endings in the *Glans clitoridis*, suggesting that it is highly sensitive and thus likely functional (Brennan et al. 2022).

In mares (*Equus caballus*), the vagina is long, constituting over 50% of the copulatory canal (Ellenberger and Baum 1915). It is joined distally by the urethra, forming UGS, which ends with a wide *Vestibulum vaginae* and vulva. The clitoral corpus is 2 cm wide and projects 6–8 cm into the ventral edge of vulva. It is constituted of joined *Corpora cavernosa*. Proximally, these continue on each side and connect by a weak *M. ischiocavernosus* (syn. *Erector clitoridis*) to the ischium. At the clitoral apex is a 2–3-cm large glans of erectile tissue, which shows a dorsal grove. The clitoris is incompletely covered by a preputial fold. Lateral to vestibular walls are two additional erectile bodies, the ellipsoid bulbs (Kobelt 1844).

Cattle and European Bison (*Bos taurus* and *Bison bonasus*) have similar anatomy, but the sizes in bison are somewhat smaller. The cow vagina is 20–28 cm long (16 cm in bison) and very wide, covered by stratified squamous epithelium, and opens caudally into 10–14 cm long *Vestibulum vaginae*. The vestibulum is covered by a stratified squamous epithelium of a few strata. Urethra opens into a wall of a large blind sack on the ventral wall of the vestibulum, so-called suburethral diverticulum (*Diverticulum suburethrale*; Ellenberger and Baum 1915). The free corpus clitoridis has a shape of a flat cone 1–2 cm long, with glans 0.5 cm wide. It is situated in the vestibulum and clearly visible on the ventral commissure of the vulvar lips. *Corpora cavernosa* are long (10–12 cm), but thin and end as *Corpora fibrosa*, attached to *ischiocavernosus* muscle. Prepuce and frenulum are described but no bulbs or baubellum (Olbrych and Szara 2011).

In sows (*Sus scrofa*), similar to cows, the urethra joins a long vagina (10–12 cm in adults), at the suburethral diverticulum, which is less prominent than in Bovines. The subsequent UGS is 7–8 cm long, ending in vestibulum and vulva. The clitoris reaches into the vestibulum by 3–4 mm, however, its overall size (including *Crurae*) is relatively large, up to 8 cm in adults. Small erectile bodies depicted latero-ventrally in the vestibular wall (Ellenberger and Baum 1915) are likely bulbs, described as small and olive-shaped by Kobelt (1844).

Carnivores are a highly diverse group with respect to clitoral anatomy, erectile bodies, and perineal muscles. The anatomy of the lower reproductive tract is less variable and consists of a mostly long vagina and UGS, variation of which depends on the structure of external parts.

In the dog (*Canis lupus familiaris*), the *Corpora spongiosa* (vestibular bulbs) are sheet-like cavernous bodies originating dorsally at the perineal membrane and extending caudally, forming the lateral walls of the vestibulum. CS are particularly large and crescent-shaped in dogs (Kobelt 1844). They join distally at the midline to form the vestibular body, which partially supports the ventral portion of urethra and finally forms the *Glans clitoridis*. A second pair of erectile bodies, *Corpora cavernosa*, also attaches to the perineal membrane and runs along the vestibular wall. These too join distally to form the large body of the clitoris (3–4 cm), together with the CS. A suspensory ligament is present, which attaches the clitoris to the pubis (Ellenberger and Baum 1915; Hall et al. 2019). Homologous erectile bodies arise also in male dogs, however, there they join immediately to form penile shaft, while in female dogs they run pairwise in the walls of vestibulum and vulva, before joining as *Corpus clitoridis*. The *Corpus clitoridis* emerges within the vestibulum cranially from the vulvar orifice, ventral to the vaginal and urethral orifice into the vulva. Urethral orifice also opens separately, ventral to the vaginal orifice into the vestibulum.

Domestic cat's (*Felis cattus*) clitoris is described as small and protruding in the ventral part of the vulva. It contains a cartilaginous *Os clitoridis* (Ellenberger and Baum 1915). The cat has a normal cervix, transitioning into a very tight and nonelastic vagina of 1–2 cm, which is followed caudally by a UGS of a similar length, ending in vulva (Watson and Glover 1993).

The male-like external morphology of the female Spotted hyena (*Crocuta crocuta*) represents a notable extreme among carnivores [for excellent detailed accounts and further literature see Cunha et al. (2003, 2014)]. This anatomical extreme is specific to only one among four hyaenid species. In this species, internal organs are relatively unremarkable, with the exception of the ovary which has an androgen-producing (testis-like) tissue. Historical reports of lacking vagina and cervix (Watson 1879) have been corrected in the subsequent work (Matthews 1939, Wells 1968; Cunha et al. 2003). Mathews, and later confirmed by Cunha, described histologically distinct vagina. Cervix is inconspicuous externally and is represented by a very short transitional zone between simple columnar uterine epithelium and the stratified epithelium of the vagina. Cunha et al. (2003) describe large cystic lumina as a distinct feature of cervix. The urethra joins with the genital canal still within the pelvic cavity, and the resulting UGS, lined by the urethral-like epithelium, is enclosed and exiting the body through a long penis-like clitorial shaft. As the orifice of the pendulous clitoris is, therefore, the only access to vagina, The Spotted hyena copulates and gives birth through the clitoris. Despite great similarity, the phallus is still sexually dimorphic in structure, including its length (in particular when erect) and circumference, the position of the urogenital orifice, and of some of the erectile bodies. Importantly, the clitoral UGS is not embedded in the CS as is the case in the penile UGS of Spotted hyena males. Interestingly, the hyena clitoral anatomy is not explained by increased androgen secretion during its development. Testosterone indeed is produced in the female fetal ovary, but only in developmental stages after phallic growth. Accordingly, experimental treatment with anti-androgens during fetal development does not reduce the growth of female phallus, rather it feminizes male fetuses. This suggests that androgen is responsible for the male-specific aspects of the phallus (thinner urogenital meatus, differences in shape of the glans, and so on), but not for its overall size. The formation of the long penile clitoris in Spotted hyena is thus androgen-independent (Cunha et al. 2014).

The description of the female reproductive tract of the raccoon (*Procyon lotor*) reports the presence of a clearly defined cervix, which transitions into the vagina, the latter measuring approximately 4 cm in length. At the height of urethral meatus the vaginal part is separated from the UGS by a fold of mucous membrane, attached to the entire circumference of the canal. The UGS measures approximately 5 cm in length. The external orifice is bounded laterally by labia, forming a prominence at the top of which is the orifice. The clitoris and its position are not described in this work (Watson 1881), and a later work only describes variation in presence of the *Os clitoridis* (Sanderson and Nalbandov 1973).

The group of Glires (roughly rabbits and rodents) includes several well-studied species, such as the cottontail rabbit, domestic rabbit, mouse, rat, hamster, chinchilla, nutria, beaver, ground squirrel, and the naked mole rat. Glires includes a large number of species in which the urethra opens into the urogenital tract either very close to its external orifice, into the flat vestibulum, or as a separate orifice. Correspondingly, the UGS forms a short vestibulum, or is missing. According to Meczynski (1974), there is no notable UGS in the following rodents: rat (*Rattus rattus*), house mouse (*Mus musculus*), golden hamster (*Mesocricetus auratus*), and Nutria (*Myocastor coypus*). An intermediate stage (flat vestibulum) is described for Guinea pig (*Cavia porcellus*), and Eurasian beaver (*Castor fiber)*, and the urethra opening high up in the reproductive tract is described for Lagomorphs, suggesting a phylogenetic progression as we will see in a later section.

A detailed study of Cottontail rabbit (*Sylvilagus floridanus*) reveals the urethral meatus 2–4 cm caudally from the cervix, forming a short vaginal segment and a relatively long UGS. (Elchlepp 1947, 1952; and the references therein). The clitoris is described as very similar to penis, only smaller and partially mobile. Internally, it consists of two *Corpora cavernosa*, which fuse distally into the clitoral shaft. On the dorsal side the surface of the shaft is not fully fused but rather forms a furrow, continuing from the urogenital vestibule. The clitoris is incompletely enveloped in prepuce and extends beyond the vulva. The internal *Corpora cavernosa* are attached laterally to the margins of the ischium. Domestic rabbit (*Oryctolagus cuniculus domesticus*) is described as similar in all above aspects (Elchlepp 1947). Perineal muscles *M. ischiocavernosus* and *M. bulbospongiosus* are present and have been studied in Chinchilla-breed rabbit doe (Cruz et al. 2002).

Meczynski (1974) studied external genitalia in two species of ground squirrel (*Spermophilus citellus* and *Spermophilus suslicus)* during estrus. In these, the UGS is broadened to a vestibulum and forms the caudal part of the genital canal, measuring about a third of the canal. Urine flows in a fold in the ventral wall. Small *Glans* and *Corpus clitoridis* appear on the ventral wall at the end of vestibulum (Meczynski 1974). The *Glans* is covered by a folded mucus membrane with a stratified epithelium. *Corpora cavernosa* are paired and join into the *Corpus clitoridis. The Os clitoridis* is well-developed in ground squirrels.

In Eurasian beaver (*C. fiber*), the *Corpus clitoridis* is long, projecting into the vestibulum, and embedded in a preputial fold. It is connected via *Crurae* to the ischium (likely by the *M. ischiocavernosum*). The urethra joins the vagina to form a short flat UGS (vestibulum), which opens with rectum into what appears to be secondarily formed *pseudocloaca* (Gienc and Doboszynska 1972; Doboszynska 1978).

Mouse genitalia are among the best studied mammalian genitalia (e.g., Rodriguez et al. 2011; Weiss et al. 2012; Cunha et al. 2018, 2020). In this species, similar to moles, the externally visible part is the prepuce, the skin pocket embedding the clitoris (or penis), while the *Corpus clitoridis* is located deep within the prepuce, which also embeds the urethra running ventrally to the *CC*. The vagina lies dorsal to this structure and opens in a separate orifice. The clitoris is thus located immediately ventral to the urethra within the prepuce. The anatomy of the mouse *Corpus clitoris* is similar to that of the penis, but smaller in size, lacks spines, has a smaller bone, and lacks the mobility relative to the prepuce due to its dorsal attachment. Importantly, the mouse (and rat) perineum appears to have only vestigial *M. ischiocavernosus and M. bulbospongiosus* (McKenna and Nadelhaft 1986).

Mole rats are another rodent group with well-studied genitalia, showing species-specific modifications in both sexes. Seney and coauthors (2009) compared clitoral and perineal anatomy in three African mole rat species: the naked (*Heterocephalus glaber*), the Damaraland (*Fukomys damarensis*), and the silvery (*Heliophobius argenteocinereus*) mole rats. The general structure of the clitoris and its position in these species is similar to that in the mouse. The clitoral shaft consists of two *Corpora cavernosa* and runs parallel and ventral to the urethra, which is separate from the vagina and lies ventral to it. Females of all three species lack CS or bulbs, whereas these are present in silvery and Damaraland mole rat males. The authors also describe the perineal muscle, in particular the *M. ischiocavernosus* and the urethral muscle. *M. ischiocavernosus* size correlates positively with the changes in size of the male phallus. It is smallest in naked mole rat and largest in Damaraland mole rat females. The urethral muscle is largest in the naked mole rat females. These species exhibit substantial penile size differences, while clitoral interspecific size differences are small, leading to a changing degree of external sexual dimorphism, and even its loss in the naked mole rats. Interestingly, while lack of dimorphism in most species is due to masculinization of female genitalia, in the naked mole rat the lack of dimorphism arises by the feminization of the male external genitalia.

In Spix's yellow-toothed cavy (*Galea spixii*, Caviidae, dos Santos et al. 2014), a simple vagina ends in the vestibulum, without distinct UGS. The vagina periodically develops a vaginal membrane covering the vaginal orifice during diestrus. The clitoris is located in the vestibulum and is traversed by the urethra. Dos Santos and collaborators also note the presence of ischiocavernosus muscle.

Red-rumped agouti (*Dasyprocta leporina*) manifests a long vagina (6 cm) lined with stratified epithelium changing its keratinization during the estrus cycle as in other rodents. The vagina opens directly to the vulva. There is no UGS or vestibulum, as the vagina and urethra open in separate external orifices as also seen in many other rodents. The vagina undergoes membrane closure during diestrus, like in the Guinea pig. The clitoris is described as prominent (if small) and conical, covered by a skin fold (likely prepuce), and manifesting at the sides of its apex two keratinized hook-like structures (spicules), between which lays urethral opening—implying that the clitoris is traversed by the urethra. Spicules change during the cycle, enlarging during estrus. Labia majora are reduced in this species (de Oliveira et al. 2019).

Of the tree shrews (Scandentia) the details could be found in the description of *Tupaia splendidula* (*ferruginosa* at the time) by Jones (1917). Jones describes a 7-mm long vagina in adult females, and the urethra joining the reproductive tract approximately half-way between the cervix and the vulva, forming the 9-mm long UGS. External genitalia manifest a prominent prolonged vulvar slit of 7 mm. The *corpus clitoridis* is small and situated median to the labia that form the edge of the slit. There is no description of the internal parts or histology of clitoris or the corresponding muscle, but the external genital structure resembles in type the primates rather than rodents.

### Primates

While the visible parts of external genitalia have been studied in many primate species in the context of behavioral studies, less information could be found on non-visible parts in nonhuman primates, apes in particular. Noted clitoral diversity in primates is predominantly due to hypertrophied clitorides in several species of lemurs and NWMs. The length of the clitoral corpus, which can be observed in the wild, received much attention, whereas there is little comparative histological detail or information on urogenital tract. It is important to note that hypertrophied clitorides are not a common occurrence in primates in general, but rather exceptions, which have been featured disproportionately often. In contrast, comparably detailed accounts of the common small clitorides, are rare.

An example of anatomical extreme is the clitoris of the Ring-tailed lemur *(Lemur catta)*. This species has a long exposed external *Corpus clitoridis*, which is traversed by the urethra (Drea and Weil, 2008). In this case, the urethra does not fuse with the genital tract to form an UGS. It becomes encased by the clitoris, whereas the vagina opens separately at the base of the clitoral corpus. The clitoris shows no CS along the urethra or at the clitoral base in the form of bulbs. An *Os citoridis* is present, the glans is elongated, and the *Crurae* run along the pubic rami and attach via *M. ischiocavernosus* and thick fascia to the ischium. Apparent *bulbospongiosus* muscle could not be found, however, a thin muscle fiber medially to *Crurae* could be its homologue (Drea and Weil 2008). It is likely that a hypertrophied clitoris is rare in prosimians, but we encountered little relevant information on this group.

Hypertrophied clitoral parts are also encountered in five genera of the NWMs: Ateline genera *Ateles, Brachyteles, Lagothryx*, and capuchin monkeys *Cebus* and *Sapajus*; (Dixson 2012). The clitoris of Spider monkey *(Ateles geoffreyi*) is large with a wide shallow grove along the ventral surface and covered by a prepuce (Wislocki 1936). It is indeed longer than the male penis and is variable in length and coloration within the species. A total of three species of Squirrel monkey, *Saimiri macrodon, S. cassiquiarensis*, and*S. vanzolinii*, with the enlarged clitorides of conical shape and with apical glans have been described by Lopes and coauthors (2017). The clitoris is covered by the preputial hood, continuous with the ventral rim of the labia majora. The vagina is relatively long and opens into the vestibulum. No information is provided about the position of the urethral meatus.

In Tufted Capuchin monkeys (*Sapajus apella*), the external clitoris has been described as a ventrally grooved elongated shaft, which reaches beyond the *Labia majora* to the outside of vulva and is enveloped in a preputial fold. The apex of the shaft expands to form the *Glans*. The elongated shape and visibility of the clitoris resemble the penis, in particular in the early developmental stages (Carosi et al. 2020). Intramembranous bone as well as CC have been described. The urethral meatus is located at the base of the clitoris in the vaginal vestibulum (ca. 2.5 cm long; Lima et al. 2015). Also in howler monkeys (*Alouatta palliata*), the clitoris is described as elongated and shallowly grooved (Wislocki 1936).

George B. Wislocki's general overview of the “simian” (platyrrhines, catarrhines, gibbons, and apes) external genitalia of the (wild-caught) animals from various collections presents a more balanced impression of New World monkeys (NWM) genital anatomy (Wislocki 1936). Wislocki describes a minute *Glans clitoridis* and large *Labia majora* in Geoffroy's marmoset [*Callithrix (Oedipomidas) geoffroyi*], as well as in Squirrel monkey *(Saimiri oerstedii*), Capuchin monkey *(Cebus capucinus*), and Night monkey *(Aotus zonalis*), in the latter with a well-developed prepuce. Similarly, Hill (1959) reports in Goeldii's marmoset (*Callimico goldii)* a small external clitoris with bifid glans which is visible at the extreme ventral edge of the vulva. This species has a keratinized vagina of about 3 cm, covered with papillae, and ending in a vestibulum. On the ventral side of the vaginal/vestibular junction rises an elevation below which there is a urethral orifice (Hill 1959). A recent account of external genitalia in the Lion tamarin [genus *Leontopithecus* (Porto et al. 2010)] also reports a small and immobile clitoris. The glans and short corpus are visible at the ventral edge of the vestibule as they reach out between the labiae. A short prepuce and frenulum can be identified. Histology shows a U-shaped clitoral corpus cross-section with a groove on the dorsal side and a fused CC. The vagina opens into the vestibulum. There is no explicit information about the exact location of the urethral meatus, however, it is clear that the urethra does not traverse the clitoris (Porto et al. 2010).

In Old World monkeys (OWM), the female external genital orifice is located close to anus, between the ischial callosities (as opposed to male genitalia, which are close to the pubic bone, thus extending the anogenital distance). This is in contrast to NWM, where the distance between the anus and the urogenital apparatus is sexually monomorphic. Wiedersheim (1908) further points out that OWM are distinct from both NWM and humans in that their vagina is relatively shorter, that is, the urethra joins the reproductive tract more cranially toward the cervix, generating a relatively longer UGS. The clitoris of OWM is described as relatively small in Rhesus macaque (*Macaca mulatta*) and situated in the ventral part of vestibulum. It is surrounded by a delicate prepuce and *Labia majora*, but lacks a frenulum (Wislocki 1936).

Wislocki also briefly describes the external genitalia of apes, which have a distinct protruding external clitoris of moderate size and vulva surrounded by two fatty crescent cushions, likely *Labia majora* (the latter has been long disputed in apes). In Black-handed gibbon *Hylobates agilis*, the prominent sub-conical clitoris is clefted, reaching onto the ventral edge from the vulva and surrounded by the prepuce (Wislocki 1936). Dahl and Nadler (1992) describe the frenulum in White-handed gibbon (*Hylobates lar*) and show a separate urethral orifice positioned in the vulva on the urethral prominence, dorsally to the clitoris and ventrally to the vaginal tube, thus lacking a tubular UGS. Authors also observed the menarcheal changes, mostly as enlargement of the labiae and external clitoral shaft and glans.

In Chimpanzee (*Pan troglodytes*), the clitoris is visible, reaching out from a very short vulva, and has a flattened form. The clitoris and genitalia of Orangutan (*Pongo sp*.) are described as similar to those of Chimpanzee. The mountain gorilla (*Gorilla beringei*) manifests a conspicuous, protruding clitoris with a well-developed prepuce, but without frenulum (Wislocki 1936).

Human female genitalia are described in various general and specialized texts in detail (e.g., O'Connell et al. 2005; Puppo 2013; Di Marino and Lepidi 2014). Particularly remarkable is the early detailed description of the clitoris by Kobelt (1844), and the accompanying extraordinarily detailed anatomical drawings. Kobelt paid particular attention to vestibular bulbs, the paired erectile bodies, *Corpora spongiosa*, positioned laterally to the vaginal walls, then fusing along the *Corpus clitoridis*, and connecting the bulbs with the glans. He also describes the muscles surrounding the bulbs and vagina as a so-called *M. constrictor cunni* (also sometimes called *M. constrictor vaginae*), which possibly refers to the *M. bulbospongiosus*. With the current data, the presence and evolution of bulbs remains an intriguing, yet open question.

The second erectile body, the CC, forms the bulk of the phallic shaft. It continues proximally as two separate *Crurae*, which attach to the pubic bone with *M. ischiocavernosus* in both sexes, and this muscle is described as not smaller in human females than in males (O'Connell et al. 2005; Di Marino and Lepidi 2014).

## Evolution of associated features

### Os clitoridis

The phylogenetic distribution of *Os clitoridis* (baubellum) has recently been reviewed extensively in comparison with the *Os penis* (baculum; Lough-Stevens et al. 2018; Spani et al. 2021). The *Os clitoridis* appears more variable than *Os penis* within but also across species. Across mammals, the phylogenetic distributions of the presence/absence of the male and female phallic bones show strong correlation between sexes. Lineages and species that lost the baculum, generally also lost the baubellum. As the clitoral bone is usually developed to a lesser extent, a small reduction in a species may be expected to lead to a loss in females sooner than in males, which also contributes to higher observed interspecific variation. Indeed, the general pattern is that in clades, where there is a loss of penile bone in several species, there is loss of the clitoral bone in an even greater number of species. Interestingly, there is a single exemption from this pattern in the data presented in Lough-Stevens et al. (2018), in which *Os penis* was lost, but *Os clitoridis* was maintained. This is the case in the European mole (*Talpa europea*), a species known for presence of ovotestes and enlarged genital parts (however, detailed analysis of whether these enlarged parts indeed represent clitoral body or prepuce is, to our knowledge, missing).

### Muscles and ligaments

The mammalian pelvic floor includes many muscles, ligaments, and fasciae, involved in a large number and diversity of pelvic functions, such as locomotion, sphincter, and reproductive functions, but also the anchoring and support of inner organs. Many ligaments of the urogenital system are dimorphic between the sexes due to the dimorphism of the urogenital system they support, but also due to the divergent functionality, e.g., pregnancy. Rather than addressing the whole complex, however, in this review we report the information about those muscles of the pelvic floor which directly relate to genital function. A comparative review of the muscles of pelvic floor would, however, be strongly needed for functional understanding. Here, most attention will thus be paid to two muscles, *M. ischiocavernosus* and *M. bulbospongiosus*.

*Musculus ischiocavernosus* connects the *Crurae* of the phallus with the ischium, and likely serves to stabilize erection in males. A second muscle, *M. bulbospongiosus*, runs circular around the basis of the penile/clitoral shaft (externally to the bulbs, if present) and is thought to maintain blood pressure, similarly thought to support male erection. The evolution of both muscles has been addressed by several classical anatomists. Gegenbaur (1898), followed by Eggeling (1939), describe two groups of striated skeletal muscles of the mammalian pelvic floor, the superficial (sphincter-related) and the deep group (i.e., coccygeus muscles), both of which are thought to have originated from different portions of the tail muscles of early vertebrates and subsequently became increasingly associated with the pelvis. The superficial group of muscles gave rise to sphincter cloacae, present (itself or its derivatives) in all amniotes, but not in amphibians. As the urogenital and rectal tracts became increasingly separated in marsupials and eutherians, the cloacal sphincter became gradually modified to form anal and urogenital sphincters. Various intermediate forms can be observed in marsupials and even some female eutherians, in which anogenital distance is very short. For example, in cats, the sphincter partially encircles either the anus or UGS, but some of the outermost fibers are common to both (Eggeling 1939). *Musculus ischiocavernosus* and *M. bulbospongiosus* are thought to both be derivatives of the *M. sphincter cloacae*. The precursor of the *M. ischiocavernosus* likely formed as a subset of fibers of the sphincter that attached to the wall of the cloaca and to the ventral rim of ischium, before the separation between rectum and UGS occurred. Concordantly, Gerhardt (1939), drawing on rich comparative work across vertebrates, reports that clitoral and penile roots are attached to the pelvic bone by the *M. ischiocavernosus* in all eutherians and some marsupials. For example, in female Quoll (*Dasyurus*; marsupial), cloaca is associated with *M. ischiocavernosus*, and Eggeling (1939) suggests that the cloacal wall may already include precursors of clitoral erectile bodies in this species. Indeed, in males, *Corpora cavernosa* (in *Crurae penis*) are described already in marsupials. In Eutherian females with UGS such as felids, *M. ischiocavernosus* attaches to the tissue just ventral to the urogenital sphincter, where also *Corpora cavernosa* appear. In contrast, *M. bulbospongiosus* likely arose by muscular fibers separating from the external urogenital sphincter muscle after the urogenital and anal tracts had diverged (Eggeling 1939).

Like for other internal parts, detailed descriptions of the perineal muscles are rare, with some exceptions. Such is the description of domestic dog by Hall and colleagues (2019), who studied the male–female homologs in external genitalia. Based on the attachment sites and relative position to erectile bodies, they suggested a unified nomenclature enabling comparison with other species. For example, they suggested that the “constrictor vulvae” and “retractor clitoris” are a continuous muscle and together represent a female homologue of male *M. bulbospongiosus*. Female “*Constrictor vestibuli “”* on the other hand is reported here to be homologous to male *M. ischiocavernosus*. Such comparative work contributes considerably to evolutionary understanding, as species- (and sex-) specific nomenclature often hinders recognition of evolutionary continuities.

## Phylogenetic reconstruction of clitoral traits

**Fig. 4:**
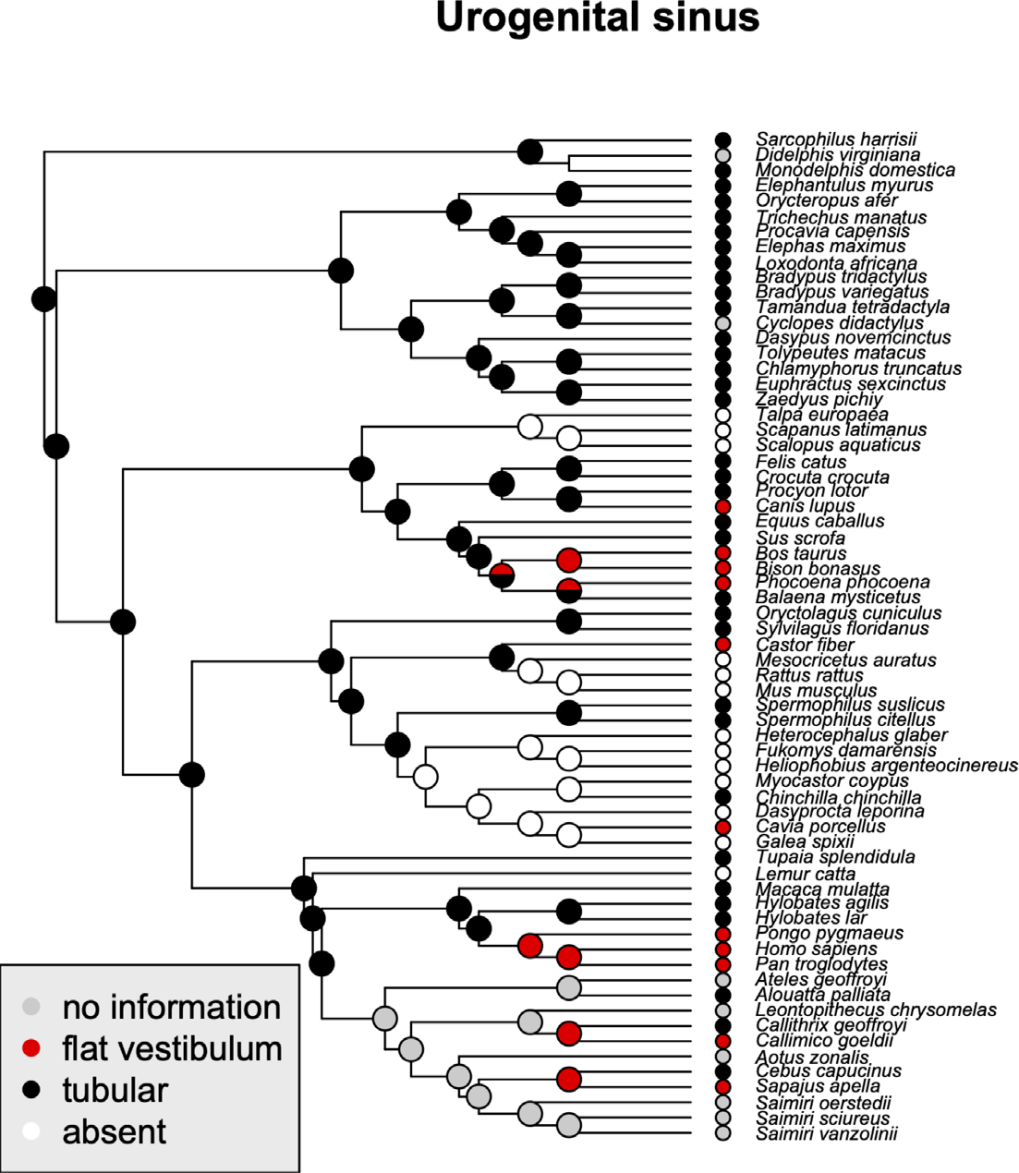
Phylogenetic distribution of different types of UGS and their inferred ancestral states. Note that tubular UGS may still end in the broadened vestibulum. It can also be represented only by a flat vestibulum or be entirely missing

**Fig. 5:**
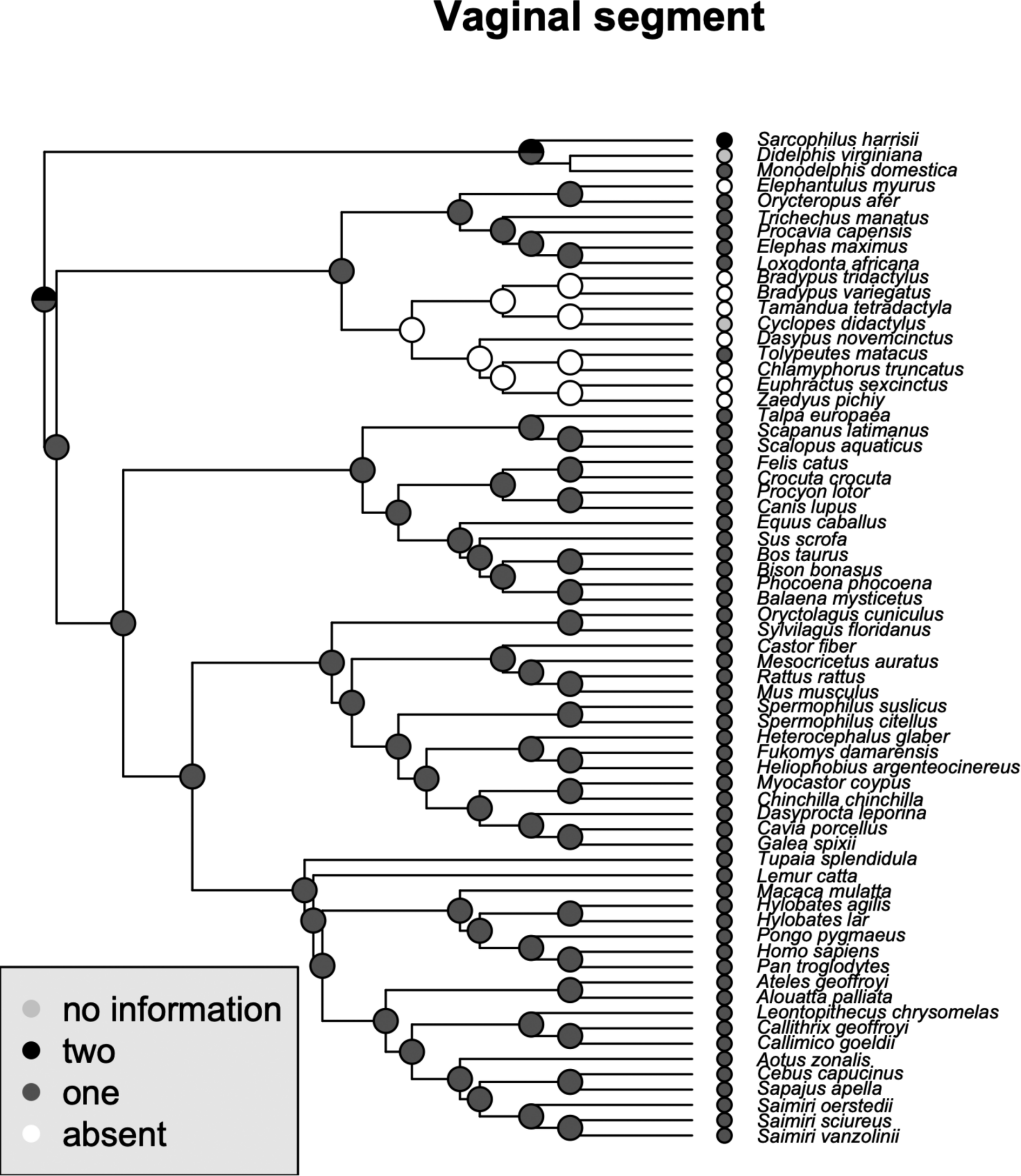
Phylogenetic distribution of different types of vaginal segments and inferred ancestral states

**Fig. 6:**
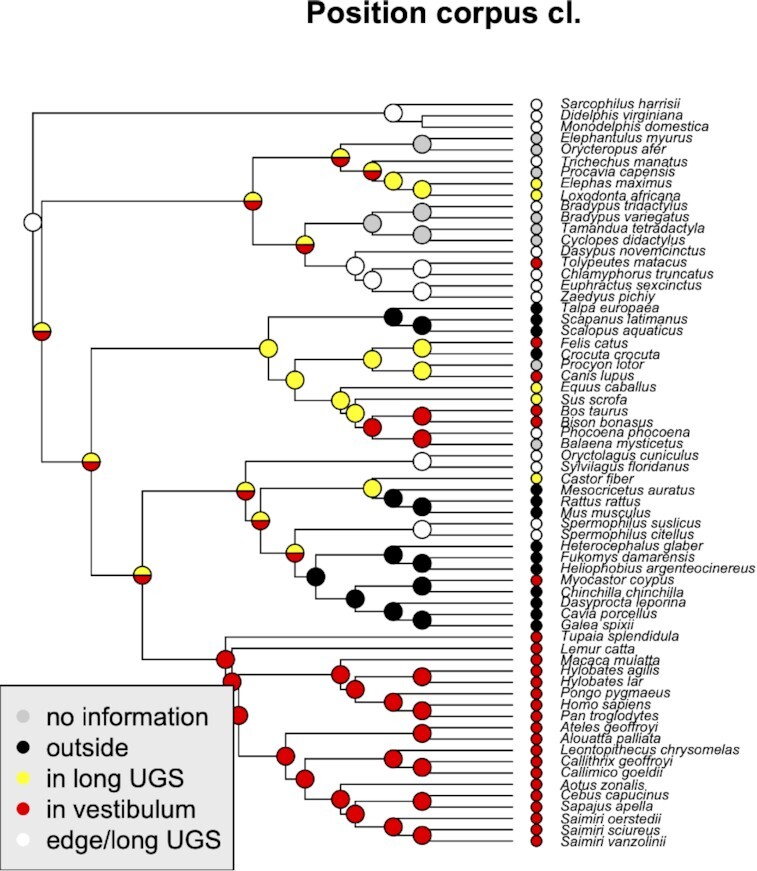
Phylogenetic distribution of different positions of *Corpus clitoridis* and their inferred ancestral states. Again, note that *“vestibulum”* can refer to vestibulum of a long tubular UGS, as well as flat vestibulum, representing the entire (short) UGS

**Fig. 7:**
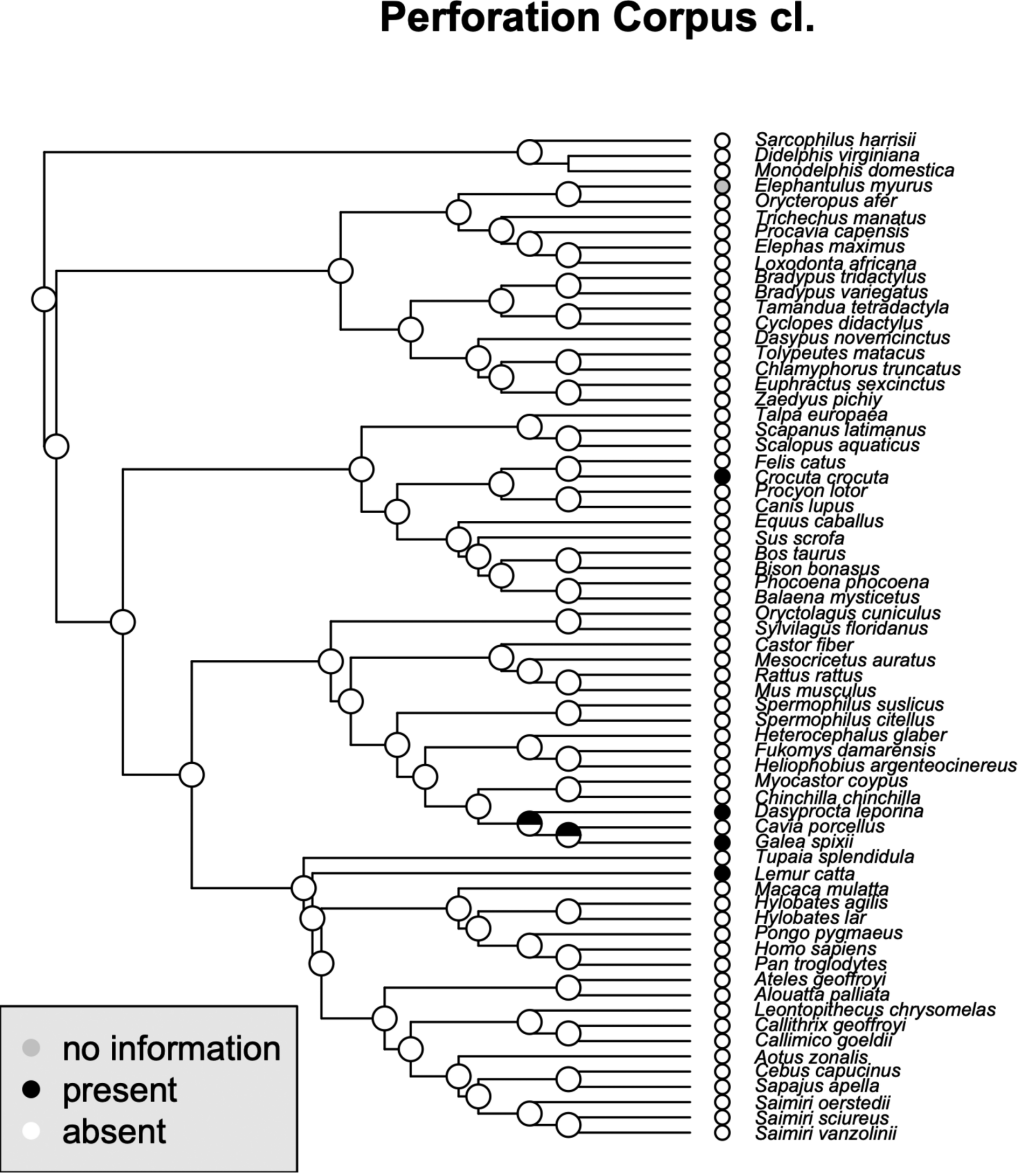
Phylogenetic distribution of perforated corpus clitoridis and their inferred ancestral states. Note the independent origins of the structure, corresponding to the anatomical analysis

**Fig. 8:**
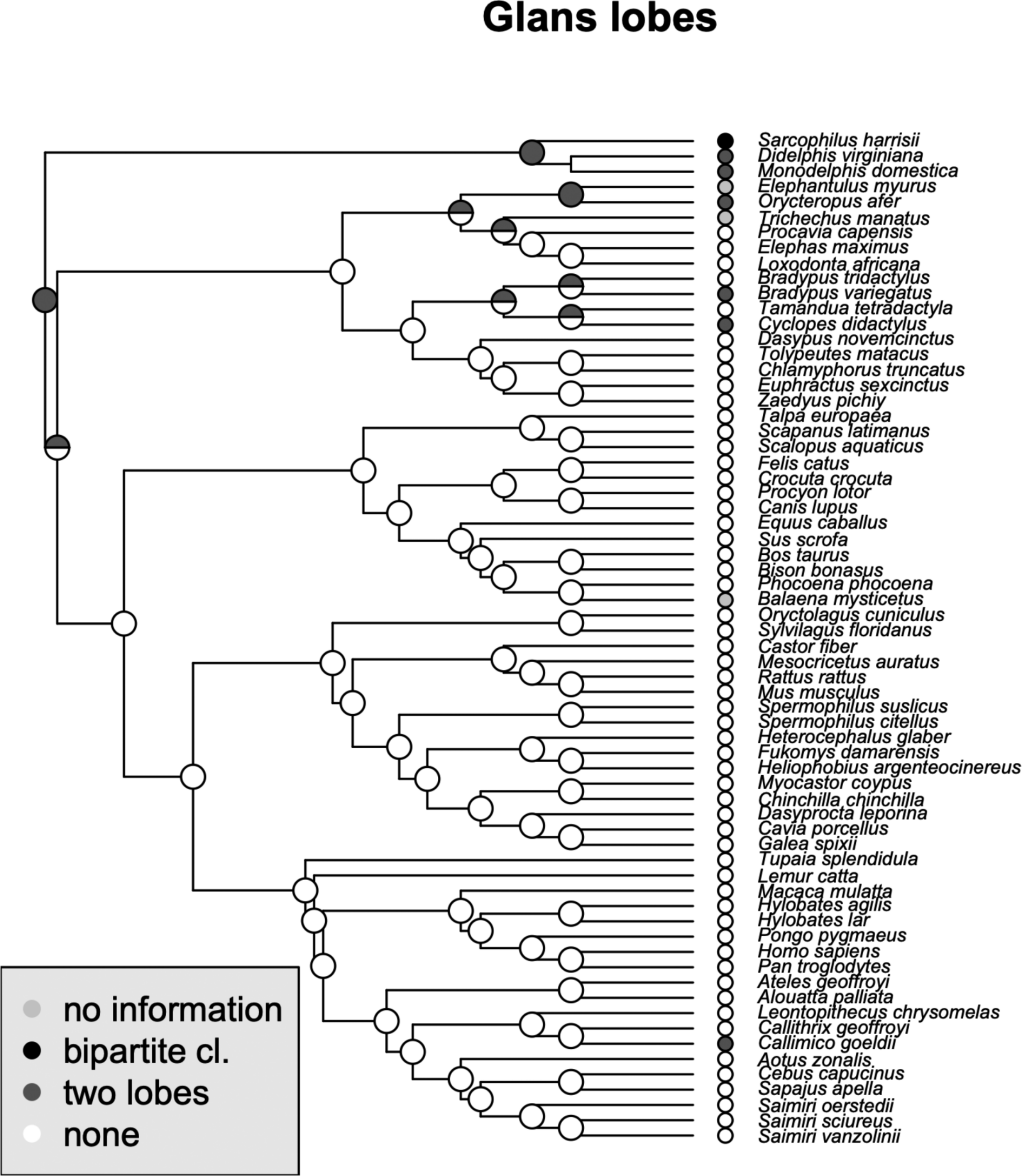
Phylogenetic distribution of glans lobes and their inferred ancestral states. Bipartite or paired lobes are seen in early branching eutherian mammals

**Fig. 9:**
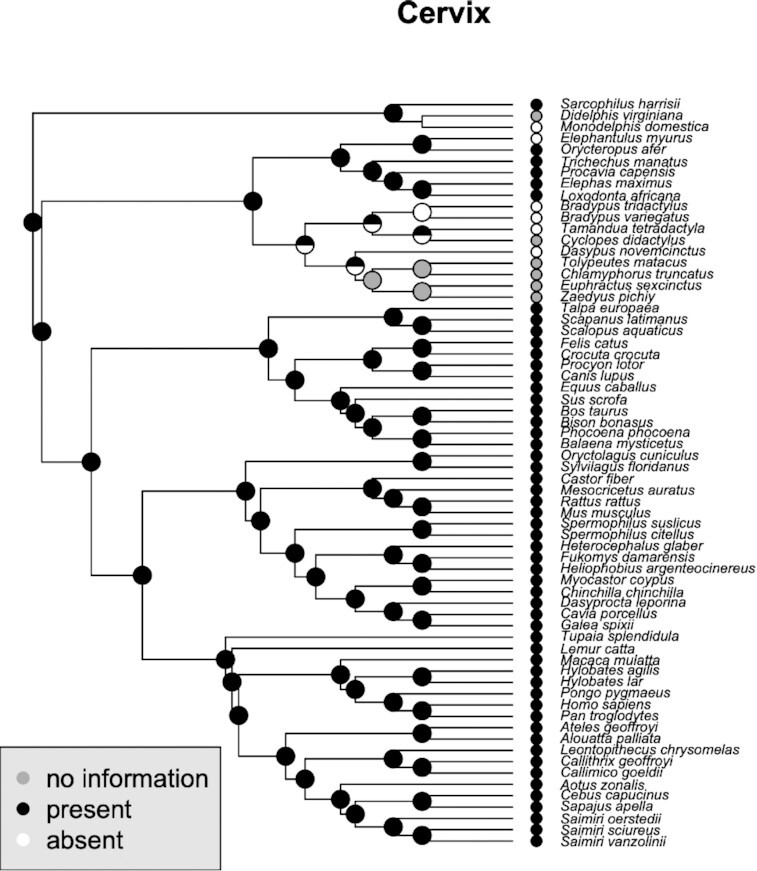
Phylogenetic distribution of cervix and the inferred ancestral states. This trait also shows greatest variation in the early branching eutherian mammals

With the information summarized above, we attempted a phylogenetic reconstruction to reveal the evolutionary changes in several characters (Figs. 4–9). We used Maximum parsimony to infer the ancestral states [R-packages Ape, v.5.5, (Paradis and Schliep 2019) and phytools, v.0.7–90 (Revell 2012)]. The tree topology is generated using Vertlife (http://vertlife.org), however, the branches are scaled for illustrative purposes and do not reflect temporal information. The character states and their coding are provided in Supplementary Table S1. In this section, we briefly discuss the results.

### UGS

The UGS, i.e., the genital canal posterior to the confluence of urethra and vagina, is highly variable in its length, if present at all. The phylogenetic reconstruction shows that a lengthy tubular type likely is the ancestral eutherian condition (Fig. 4), with independent shortening and even loss in different clades. Typical is the loss in many rodents, but also among talpids (Eulipotyphla). A reduction to flat vestibulum is common among great apes and NWMs, as well as independently in some hoofed animals, such as the cow.

### Vaginal segment

We defined the vaginal segment as the portion of the reproductive tract between the cervix and the urethral meatus (Fig. 5). As xenarthrans and some afrotherians lack a cervix, and the uterus ends in a long uterovaginal canal that is followed by the urethral meatus, they also lack a vagina. The phylogenetic reconstruction shows that the absence of the vaginal segment is limited to this clade.

### Position of the *Corpus clitoridis*

The position of the *Corpus clitoridis* is highly variable among clades (Fig. 6), and consistent within major clades. As it is defined here, based on the relation to other variable traits, UGS in particular, this is not surprising. Thus in rodents and independently in talpids, the species which lack UGS, the corpus clitoridis protrudes on the outside of the body, whereas it is located at the ventral edge of vestibulum in primates. Interestingly, ancestrally the *C. clitoridis* is inferred to be located at the caudal end of a tubular UGS and this condition is maintained in several eutherian species, above all in Xenarthrans. The *Corpus clitoridis* appears to have been repositioned towards a deeper location in the UGS in Laurasiatherians.

### Traversed clitoris

The phylogenetic reconstruction recapitulated what was already discussed in the previous section (Fig. 7). Traversed clitorides appear in several species; however, these species are not closely related and, hence these clitoral structures have evolved independently at least three times. This is congruent with the traversing involving different ducts (i.e., either urethra or UGS) in different species, as explained above.

### Glans lobes

A bipartite structure of the *Glans clitoridis* appears to be characteristic of early branching groups of therian mammals (Marsupials, Afrotheria, and Xenarthra), and is absent in all other eutherian clades and is thus likely ancestral in therians and lost in boreoeutherians (Fig. 8).

### Cervix

**Fig. 10:**
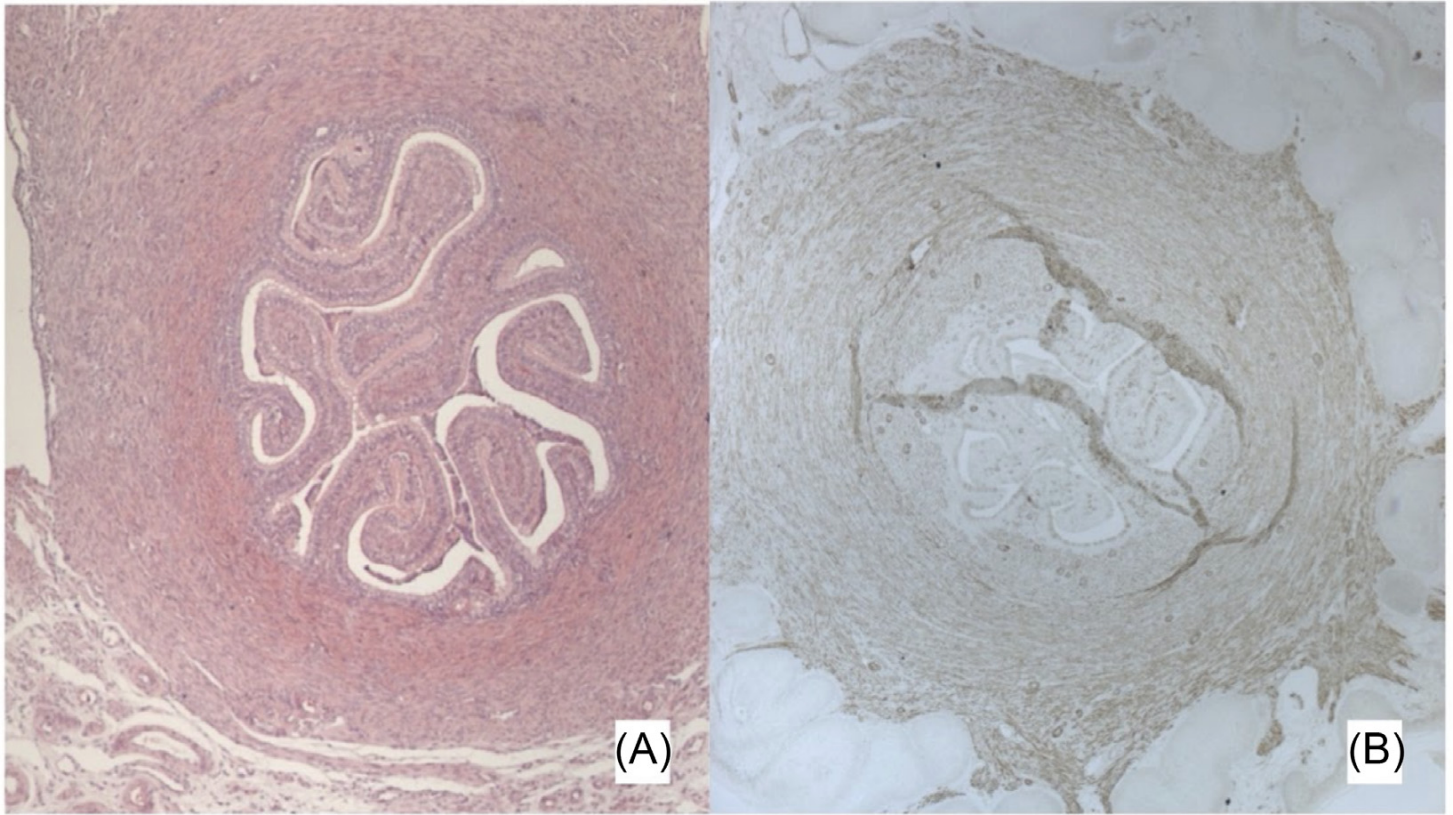
Cross-sections through the cervix of an opossum (*Monodelphis domestica*, specimen Md_13K5 from the opossum colony at Yale University). The female was non-pregnant/non-cycling (opossum females cycle only in presence of males). (**A**) Hematoxylin-eosin staining suggesting a ring of smooth muscle around the cervical canal. The latter is deeply folded. (**B**) Immuno-histochemical staining for smooth muscle actin (SMA) visualized with horseradish peroxidase reaction (brown staining). Note the ring of SMA staining around the cervical canal, supporting the notion that the opossum cervix is a smooth muscle organ similar to that of reptiles and birds. In the mouse and the human, the cervical stroma is primarily collagenous with very few smooth muscle cells

The phylogenetic reconstruction presented in Fig. 9 reflects the unusual situation of a lack of a true cervix in Xenarthra and some species of Afrotheria (elephant shrews). The inference that this situation is a derived state depends crucially on whether the cervix of the outgroup, Marsupialia, is homologous to the eutherian cervix and thus that the mammalian cervix already existed prior to the branching of Xenarthra. An observation that supports this idea is that in one of the investigated species of armadillo (southern three-banded armadillo, *T. matacus)*, a true vagina and possibly cervix has been reported (Cetica et al. 2005). Similarly, cervix is absent in at least one family of Afrotheria. Such variation in early branching lineages would otherwise have to be explained as multiple separate origins of the cervix. However, even if the marsupial cervix is homologous to that of eutherians as a structural unit, it still may have undergone a profound transformation in its histological structure. In Eutherians the cervix is mostly a collagenous connective tissue, while in the opossum it is a smooth muscle organ (Fig. 10). To clarify this question will require detailed histological study of more marsupial and afrotherian cervices and further study of the xenarthran reproductive tracts.

## Discussion and conclusion

The current review focuses on the insights obtained by compiling published information on female genital anatomy, clitoral anatomy in particular. In many cases, the referenced works will lead the interested reader to further, older literature on aspects of female genitalia. Whenever possible, for our purpose we have used the references that review older literature, and thereby establish some consensus even when diverging accounts exist; these may be either due to small sample sizes, the use of captive animals, or attraction to particularly extreme individuals. Following up on inconsistencies in original sources is often a fruitful research approach, but outside the scope of this compilation.

The insights resulting from our compilation can only be considered preliminary, due to the fragmentary information on the mammalian female reproductive tract. The aims of this review are to, next to presenting preliminary evolutionary patterns, point to weaknesses of the existing data and the many questions that remain unanswered even in the mammalian anatomy.

However, the data presently available do reveal an enormous diversity in the anatomy of the female mammalian lower reproductive tract and the genitalia. We observed that the variation is greater with respect to the external genital organs than the primary reproductive tract features (ovaries, uterus, etc.). Also, the variation appears greater in the early branching eutherian clades, in particular in Xenarthrans, than in Boreoeutheria and the later branching clades within Boreoeutheria, such as primates or rodents. In general, female external genital traits apparently possess high evolvability, manifested not only by high phenotypic variation, but also by independent origination of similar structures, using different evolutionary paths, such as is the case with hypertrophied clitoris or its associated parts. The same can also be said about the internal clitoral structures. Internally placed paired clitoral roots consisting of *Corpora cavernosa* in most cases fuse to form a single clitoral body. This fusion is likely a derived state in eutherian mammals, as the marsupial and xenarthran species show various degrees of paired external parts in adults, and even in the human clitoral corpus, the septum between the two *Corpora cavernosa* can be seen (Di Marino and Lepidi 2014). Despite scarce information, the presence of internal clitoral roots is reported consistently enough to assume that these parts are generally present in therian females, as they are in males. This appears different for the *Corpora spongiosa*, the clitoral bulbs. These inner parts associated with the clitoris are rarely described, yet few authors explicitly mention their absence, which would indicate that an attempt was made to search for them. Kobelt (1844) reports their presence in humans, rats, pigs, dogs, and horses, all the species he studied. He suggests that the *Bulbus spongiosum* are generally present in female mammals (as it is in males) but show great variation in size. It is at this point uncertain whether this part is rare, very inconspicuous, or whether a lack of focus on this region has prevented an assessment of its phylogenetic distribution. In contrast, bulbs are a very well-developed part of the human female clitoral complex (e.g., Di Marino and Lepidi 2014). While this particularly large bulb size is clearly a derived state, it is unclear when this enlargement evolved, as this structure is not described in OWM or apes.

This compilation inevitably inherits the weaknesses of the original literature. Apart from the mentioned sparsity of anatomical information and small sample sizes per species, the weaknesses include undetermined phase of the ovarian cycle at the time of collection or observation, unbalanced taxon sampling, and sometimes unclear homology relations. First, the genitalia of many mammalian females change during the reproductive period of the cycle, hence adding to variation, although the general location of anatomical structures will not be affected. Second, there is understandably a bias in favor of research on species with unusual anatomy, such as the hypertrophied clitoris of the Spotted hyena females. The bias toward these extremes should not be taken as a sign of the common occurrence of such anatomies, but rather as the lack of coverage of *less extreme* morphologies. Third, and most important, comparative work requires identification of homologous parts, which can be difficult from descriptions of the female genitalia and perineal muscles. Whereas the female external genitalia and lower genital tract are of common origin in therian females, the definitions of their parts are sometimes derived from their spatial interrelationship (e.g., UGS), from their histology (e.g., epithelium), or their function (e.g., copulatory organ). Prioritizing one of the aspects is a statement about their relative rates of evolutionary change among the aspects. For example, if the location of urethral meatus relative to reproductive tract is considered most evolutionarily stable, we can consider the part from the cervix to the meatus to be the vagina (or the vaginal segment), and the part from the meatus to the urogenital orifice to be the UGS, across all mammals. Would we encounter a species in which epithelium does not change consistently with this border, we may conclude that changes arose by epithelial migration, or the change of the epithelial morphology as adaptation to its function (squamous stratified epithelia are indicative of mechanical or other stresses as occur during copulation and not an indication of homology). Alternatively, prioritizing the histology as the most stable aspect, we may conclude that the urethra can open into the midsection of the vagina, and that we must reconsider the naming of the caudal part as UGS and, thus the criterion of the transition between the two. Similarly, if we define vagina by the function as a copulatory organ, this will include UGS in some species and not in others. In addition to these challenges, comparative approaches must overcome the presently inconsistent anatomical nomenclature across the extended history of anatomical studies.

Most of these challenges will be overcome by extending the range of research on female reproductive and genital anatomy, as appears to be well underway. In order to understand how the genital anatomy evolves in the context of its reproductive functions, it will be important to combine anatomical knowledge with neuroanatomy, physiology, and endocrinology, beyond the excellent works on genital function in humans and mice (Martin-Alguacil et al. 2008a, 2008b, 2008c).

At the very least this overview should cast doubt on the widely held view that “diverse male genitalic morphology” contrasts with “uniform female morphologies” (Eberhard 1985) and show that this perceived pattern is largely based on ignorance. It is at least unlikely that the range of genital morphologies summarized above are accidental. However, the real biological meaning of these anatomical variations will be revealed when contextualized with other aspects of female reproductive biology, sexuality and physiology (Dixson 2021).

## Supplementary Material

icac026_Supplemental_File

## Notes

From the symposium “Morphology and evolution of female copulatory morphology in Amniotes” presented at the annual meeting of the Society for Integrative and Comparative Biology virtual annual meeting, January 3–February 28, 2022.

## References

[bib1] Armfield BA , Cohn MJ. 2021. Single cell transcriptomic analysis of external genitalia reveals complex and sexually dimorphic cell populations in the early genital tubercle. Dev Biol. 477:145–54. 34033822 10.1016/j.ydbio.2021.05.014 PMC12926789

[bib2] Balke JM , Boever WJ, Ellersieck MR, Seal US, Smith DA. 1988. Anatomy of the reproductive tract of the female African elephant (*Loxodonta africana*) with reference to development of techniques for artificial breeding. Reproduction. 84:485–92. 10.1530/jrf.0.0840485 3199367

[bib3] Brennan PLR , Cowart JR, Orbach DN. 2022. Evidence of a functional clitoris in dolphins. Curr Biol. 32:R24–6. 35015987 10.1016/j.cub.2021.11.020

[bib4] Bulmer D. 1957. The development of the human vagina. J. Anat. 9:490–509. PMC1244904 13475148

[bib5] Carlson B. 2014. Human embryology and developmental biology. 5th ed. Amsterdam: Elsevier, Saunders.

[bib6] Carosi M , Spani F, Ulland AE, Scalici M, Suomi SJ. 2020. Clitoral length in immature and mature captive tufted capuchin (*Sapajus* spp.) females: a cross-sectional study. Am J Primatol. 82:e23135. 32319142 10.1002/ajp.23135 PMC7577954

[bib7] Carter AM , Goodman SM, Enders AC. 2008. Female reproductive tract and placentation in sucker-footed bats (Chiroptera: Myzopodidae) endemic to Madagascar. Placenta. 29:484–91. 18374977 10.1016/j.placenta.2008.02.009

[bib8] Carter AM , Mess A. 2008. Evolution of the placenta and associated reproductive characters in bats. J Exp Zool. 310B:428–49. 10.1002/jez.b.21216 18481267

[bib9] Cetica PD , Marcos HJA, Merani MS. 2005. Morphology of female genital tracts in Dasypodidae (Xenarthra, Mammalia): a comparative study. Zoomorphology. 124:27–65.

[bib105_1653512502046] Crichton EB , Krutzsch PH. 1987. Reproductive biology of the female little mastiff bat Mormopterus planiceps in southeast Australia. Am J Anat. 178:369–86. 3604956 10.1002/aja.1001780408

[bib10] Cruz Y , Hudson R, Pacheco P, Lucio RA, Martinez-Gomez M. 2002. Anatomical and physiological characteristics of perineal muscles in the female rabbit. Physiol Behav. 75:33–40. 11890950 10.1016/s0031-9384(01)00638-2

[bib11] Cunha GR , Liu G, Sinclair A, Cao M, Baskin L. 2020. Clitoral development in the mouse and human. Differentiation. 111:79–97. 31731099 10.1016/j.diff.2019.07.006

[bib12] Cunha GR , Risbridger G, Wang H, Place NJ, Grumbach M, Cunha TJ, Weldele M, Conley AJ, Barcellos D, Agarwal S, et al. 2014. Development of the external genitalia: perspectives from the spotted hyena (*Crocuta crocuta*). Differentiation. 87:4–22. 24582573 10.1016/j.diff.2013.12.003 PMC4069199

[bib13] Cunha GR , Robboy SJ, Kurita T, Isaacson D, Shen J, Cao M, Baskin LS. 2018. Development of the human female reproductive tract. Differentiation. 103:46–65. 30236463 10.1016/j.diff.2018.09.001 PMC6234064

[bib14] Cunha GR , Wang Y, Place NJ, Liu W, Baskin LS, Glickman SE. 2003. Urogenital system of the Spotted Hyena (*Crocuta crocuta Erxleben*): a functional histological study. J Morphol. 256:205–18. 12635111 10.1002/jmor.10085

[bib15] Dahl JF , Nadler RD. 1992. The external genitalia of female gibbons, *Hylobates* (H.) *lar*. Anat Rec. 232:572–8. 1554106 10.1002/ar.1092320412

[bib106_1653512972071] Di Marino V , Lepidi H. 2014. Anatomic Study of the Clitoris and the Bulbo-Clitoral Organ. Springer, Heidelberg.

[bib16] de Oliveira GB , de Araujo HN, Sousa RD, Bezerra FVF, dos Santos AC, de Moura CEB, Silva AR, Rocha HAD, de Oliveira MF. 2019. Morphology of the genital organs of the female red-rumped agouti (Dasyprocta leporina, Linnaeus, 1758) during estrous cycle phases and in advanced pregnancy. J Morphol. 280:1232–45. 31233245 10.1002/jmor.21027

[bib17] Dixson AF , Anderson M. 2001. Sexual selection and the comparative anatomy of reproduction in monkeys, apes, and human beings. Annu Rev Sex Res. 12:121–44. 12666739

[bib18] Dixson AF. 2012. Primate sexuality : comparative studies of the prosimians, monkeys, apes, and humans. Oxford: Oxford University Press.

[bib19] Dixson AF. 2021. Mammalian sexuality: the act of mating and the evolution of reproduction. Cambridge: Cambridge University Press.

[bib20] Doboszynska T. 1978. Histomorphology of the female reproductive system of the European Beaver. Acta Theriol. 23:99–125.

[bib21] dos Santos AC , Bertassoli BM, Viana DC, Vasconcelos BG, de Oliveira MF, Miglino MA, Neto ACD. 2014. The morphology of female genitalia in *Galea spixii* (Caviidae, Caviinae). Biosci J. 30:1793–802.

[bib22] Drea CM , Weil A. 2008. External genital morphology of the ring-tailed lemur (*Lemur catta*): females are naturally “masculinized.”. J Morphol. 269:451–63. 17972270 10.1002/jmor.10594

[bib23] Eberhard WG. 1985. Sexual selection and animal genitalia. Cambridge (MA): Harvard University Press.

[bib24] Eggeling H. 1939. Die Muskeln des Beckenausganges. In: Handbuch der vergleichenden Anatomie der Wirbeltiere, Vol. 6. Berlin: Urban & Schwarzenberg, pp. 351–74.

[bib25] Elchlepp JG. 1947. The prostate of the female cottontail rabbit, *Sylvilagus floridanus*. Anat Rec. 99:656. 18895454

[bib26] Elchlepp JG. 1952. The urogenital organs of the cottontail rabbit (*Sylvilagus floridanus*). J Morphol. 91:169–98.

[bib27] Ellenberger W , Baum H. 1915. Handbuch der vergleichenden Anatomie der Haustiere. Heidelberg: Springer.

[bib28] Enders AC , Buchanan GD. 1959. The reproductive tract of the female nine-banded armadillo. Tex Rep Biol Med. 17:323–40. 13820242

[bib29] Favoretto SM , daSilva EG, Menezes J, Guerra RR, Campos DB. 2016. Reproductive system of Brown-throated Sloth (*Bradypus variegatus*, Schinz 1825, Pilosa, Xenarthra): anatomy and histology. Anat Histol Embryol. 45:249–59. 26250652 10.1111/ahe.12193

[bib30] Flyn TT. 1910. Contributions to a knowledge of the anatomy and development of the marsupialia. No. I: the genitalia of *Sarcophilus satanicus* (female). Proc Linnean Soc New South Wales. 35:873–87.. RoySoc Serial LIN.

[bib31] Flyn TT. 1911. Notes on marsupialian anatomy II. On the female genital organs of a virgin *Sarcophilus satanicus*. Papers and Proceedings of the Royal Society of Tasmania. Hobart: University of Tasmania Library Special and Rare Materials Collection. pp. 144–61.

[bib32] Gegenbaur C. 1898. Vergleichende Anatomie der Wirbeltiere mit Berücksichtigung der Wirbellosen. Leipzig: Verlag von Wilhelm Engelmann.

[bib33] Gerhardt U. 1939. Kloake und Begattungsorgane. In: Bolk L. et al. et al.editors. Handbuch der vergleichenden Anatomie der Wirbeltiere, Vol. 6. Berlin: Urban & Schwarzenberg, pp. 267–350.

[bib34] Gienc J , Doboszynska T. 1972. Macromorphological description of the genital organs of the female beaver. Acta Theriol. 17:399–406, plates 1-2.

[bib35] Giersberg H , Rietschel P. 1968. Vergleichende Anatomie der Wirbeltiere. Jena: Gustav Fischer Verlag.

[bib36] Gredler ML , Larkins CE, Leal F, Lewis AK, Herrera AM, Perriton CL, Sanger TJ, Cohn MJ. 2014. Evolution of external genitalia: insights from reptilian development. Sex Dev. 8:311–26. 25115961 10.1159/000365771

[bib37] Gredler ML. 2016. Developmental and evolutionary origins of the *Amniote phallus*. Integr Comp Biol. 56:694–704. 27549197 10.1093/icb/icw102

[bib38] Hall MI , Plochocki JH, Rodriguez-Sosa JR. 2019. Male and female anatomical homologies in the perineum of the dog (*Canis familiaris*). Vet Med Sci. 5:39–47. 30663868 10.1002/vms3.128 PMC6376168

[bib39] Hayssen V. 2011. *Tamandua tetradactyla* (Pilosa:Myrmecophagidae). Mammal Species. 43:64–74.

[bib40] Hill WCO. 1959. The anatomy of *Callimico goeldii* (Thomas) a primitive American primate. Philadelphia (PA): American Philosophical Society.

[bib42] Hood CS , Smith JD. 1983. Histomorphology of the female reproductive tract in phyllostomoid bats. Lubbock (TX): Texas Tech Press.

[bib41] Hood CS. 1989. Comparative morphology and evolution of the female reproductive tract in macroglossine bats (mammalia, chiroptera). J Morphol. 199:207–21. 29865633 10.1002/jmor.1051990207

[bib43] Jones FW. 1917. The genitalia of Tupaia. J Anat. 51:118–26. 17103808 PMC1262795

[bib44] Kobelt GL. 1844. Die männlichen und weiblichen Wollust-Organe des Menschen und einiger Säugetiere. Freiburg: Druck und Verlag vom Adolf Emmerling.

[bib46] Langer W. 1913. Beiträge zur morphologie der viviparen cyprinodontiden. Gegenbaurs Morphol Jahrb. 47:193–307.

[bib47] Levin RJ. 2019. The clitoris—an appraisal of its reproductive function during the fertile years: why was it, and still is, overlooked in accounts of female sexual arousal. Clin Anat. 32:136–45. 10.1002/ca.23498 31691374

[bib48] Lima AR , Guimaraes SB, Branco E, Giese EG, Muniz JA, Pereira WL, da Cunha PK, Ricci RE, Miglino MA. 2015. Morphological and morphometric description of female reproductive tract of *Sapajus apella* (Capuchin monkey). Anat Histol Embryol. 44:262–8. 25091087 10.1111/ahe.12134

[bib49] Lonsky F. 1903. Beiträge zur Anatomie und Entwickelungsgeschichte des darmrohres und des Urogenitalsystemes von Hyrax. Jenaische Zeitschrift für Naturwissenschaft. 37:579–652.

[bib50] Lopes GP , Brito AB, Paim FP, Santos RR, Queiroz HL, Domingues SF. 2017. Comparative characterization of the external genitalia and reproductive tubular organs of three species of the genus *Saimiri Voigt*, 1831 (primates: Cebidae). Anat Histol Embryol. 46:143–61. 27574183 10.1111/ahe.12246

[bib51] Lough-Stevens M , Schultz NG, Dean MD. 2018. The baubellum is more developmentally and evolutionarily labile than the baculum. Ecol Evol. 8:1073–83. 29375780 10.1002/ece3.3634 PMC5773289

[bib56] McCarthy TJ , Robertson P, Mitchell J. 1988. The occurrence of *Tonatia schulzi* (Chiroptera: Phyllostomidae) in French Guiana with comments on the female genitalia. Mammalia. 52:4.

[bib59] McCrady E. 1938. The embryology of the opossum. Philadelphia (PA): The Wistar Institute of Anatomy. p. 233.

[bib60] McKenna KE , Nadelhaft I. 1986. The organization of the pudendal nerve in the male and female rat. J Comp Neurol. 22:532–49. 10.1002/cne.902480406 3722467

[bib52] Mancina CA. 2005. Pteronotus macleayii. Mammal Species. 778:1–3.

[bib54] Martin-Alguacil N , Pfaff DW, Shelley DN, Schober JM. 2008b. Clitoral sexual arousal: an immunocytochemical and innervation study of the clitoris. BJU IntBJU Int. 101:1407–13. 10.1111/j.1464-410X.2008.07625.x 18454796

[bib53] Martin-Alguacil N , Schober J, Kow LM, Pfaff D. 2008a. Oestrogen receptor expression and neuronal nitric oxide synthase in the clitoris and preputial gland structures of mice. BJU IntBJU Int. 102:1719–23. 10.1111/j.1464-410X.2008.07989.x 18793302

[bib55] Martin-Alguacil N , Schober JM, Sengelaub DR, Pfaff DW, Shelley DN. 2008c. Clitoral sexual arousal: Neuronal tracing study from the clitoris through the spinal tracts. J Urol. 180:1241–8. 18707740 10.1016/j.juro.2008.06.009 PMC2740385

[bib57] Matthews L . 1939. Reproduction of the spotted hyena (*Crocuta crocuta Erxleben*). Phil Trans R Soc Lond B. 230:1–78.. Proceedings of the Zoological Society of London B111, 289-342.

[bib58] Matthews LH. 1942. Notes on the genitalia and reproduction of some African rats. Proc Zool Soc Lond. B111:289–342.

[bib61] Meczynski S. 1974. Morphohistological structure of female genital organs in sousliks. Acta Theriol. 19:91–106. 4478856

[bib62] Meek A , 1918. The reproductive organs of Cetacea. J Anat. 52:186–210. 17103833 PMC1262833

[bib63] Morejohn GV , Baltz DM. 1972. On the reproductive tract of the female Dall porpoise. J Mammal. 53:606–8. 5074820

[bib64] O'Connell HE , Sanjeevan KV, Hutson JM. 2005. Anatomy of the clitoris. J Urol. 174:1189–95. 16145367 10.1097/01.ju.0000173639.38898.cd

[bib65] Olbrych K , Szara T. 2011. Morphology of external female reproductive organs in European bison (*Bison bonasus L*.). Eur Bison Conser Newslett. 4:55–62.

[bib66] Orbach DN , Brassey CA, Gardiner JD, Brennan PLR, 2021. 3D genital shape complexity in female marine mammals. Ecol Evol. 11:3210–8. 33841778 10.1002/ece3.7269 PMC8019040

[bib67] Orbach DN , Rattan S, Hogan M, Crosby AJ, Brennan PLR. 2019. Biomechanical properties of female dolphin reproductive tissue. Acta Biomater. 86:117–24. 30641290 10.1016/j.actbio.2019.01.012

[bib68] Paradis E , Schliep K. 2019. ape 5.0: an environment for modern phylogenetics and evolutionary analyses in R. Bioinformatics. 35:526–8. 30016406 10.1093/bioinformatics/bty633

[bib69] Pavlicev M , Wagner G. 2016. The evolutionary origin of female orgasm. J Exp Zool B Mol Dev Evol. 326:326–37. 27478160 10.1002/jez.b.22690

[bib70] Pavlicev M , Zupan AM, Barry A, Walters S, Milano KM, Kliman HJ, Wagner GP. 2019. An experimental test of the ovulatory homolog model of female orgasm. Proc Natl Acad Sci USA. 116:20267–73. 31570579 10.1073/pnas.1910295116 PMC6789565

[bib71] Perry JS. 1951. Reproduction of the African elephant, *Loxodonta africana*. J Endocrinol. 7:liii–lv. 14888793

[bib72] Pocock RI. 1924a. Some external characters of*Orycteropus afer*. Proc Zool Soc. 46:697–706.

[bib73] Pocock RI. 1924b. The external characters of the South American Edentates. Proc Zool Soc Lond. 94:983–1031.

[bib74] Porto M , Pissinatti A, Burity CHF, Tortelly R, Pissinatti L. 2010. Morphological description of the clitoris from the *Leontopithecus rosalia* (Linnaeus, 1766), *Leontopithecus chrysomelas* (Kuhl, 1820) and *Leontopithecus chrysopygus* (Mikan, 1823) (Primates, Platyrrhini, Callitrichidae). Ann Natl Acad Med. 180:1–9.

[bib75] Puppo V. 2013. Anatomy and physiology of the clitoris, vestibular bulbs, and labia minora with a review of the female orgasm and the prevention of female sexual dysfunction. Clin Anat. 26:134–52. 23169570 10.1002/ca.22177

[bib76] Revell LJ. 2012. phytools: an R package for phylogenetic comparative biology (and other things). Methods Ecol Evol. 3:217–23.

[bib77] Robboy SJ , Kurita T, Baskin L, Cunha GR. 2017. New insights into human female reproductive tract development. Differentiation. 97:9–22. 28918284 10.1016/j.diff.2017.08.002 PMC5712241

[bib78] Rodrigues AF , Santiago CS, Morielle-Versute E, Taboga SR, Beguelini MR. 2019. Morphological variation of the female reproductive organs of the bat *Artibeus lituratus* during its different reproductive phases. J Morphol. 280:1141–55. 31194263 10.1002/jmor.21006

[bib79] Rodrigues FR , Da Silva VM, Barcellos JF, Lazzarini SM. 2008. Reproductive anatomy of the female Amazonian manatee *Trichechus inunguis* Natterer, 1883 (Mammalia: Sirenia). Anat Rec. 291:557–64. 10.1002/ar.20688 18383272

[bib80] Rodriguez E Jr. , Weiss DA, Yang JH, Menshenina J, Ferretti M, Cunha TJ, Barcellos D, Chan LY, Risbridger G, Cunha GR, et al. 2011. New insights on the morphology of adult mouse penis. Biol Reprod. 85:1216–21. 21918128 10.1095/biolreprod.111.091504 PMC3223253

[bib81] Rommel SA , Pabst DA, McLellan WA. 2007. Functional anatomy of the cetacean reproductive system, with comparison to the domestic dog. In: Miller DE, editors. Reproductive biology and phylogeny of Cetacea: whales, porpoises and dolphins, Vol. 1. Boca Raton (FL): CRC Press.

[bib82] Rossi LF , Luaces JP, Marcos HJ, Cetica PD, Gachen G, Jimeno GP, Merani MS. 2011. Female reproductive tract of the lesser anteater (*Tamandua tetradactyla*, myrmecophagidae, Xenarthra). Anatomy and histology. J Morphol. 272:1307–13. 21732404 10.1002/jmor.10983

[bib83] Rubenstein NM , Cunha GR, Wang YZ, Campbell KL, Conley AJ, Catania KC, Glickman SE, Place NJ. 2003. Variation in ovarian morphology in four species of New World moles with a peniform clitoris. Reproduction. 126:713–9. 14748690 10.1530/rep.0.1260713

[bib84] Sanderson GC , Nalbandov AV. 1973. The reproductive cycle of the raccoon in Illinois. Illinois Nat Hist Surv Bull. 31:29–85.

[bib85] Sanger TJ , Gredler ML, Cohn MJ, 2015. Resurrecting embryos of the tuatara, *Sphenodon punctatus*, to resolve vertebrate phallus evolution. Biol Lett. 11:20150694. 26510679 10.1098/rsbl.2015.0694 PMC4650183

[bib86] Schutz NG , Lough-Stevens M, Abreu E, Orr T, Dean MD. 2016. The baculum was gained and lost multiple times during mammalian evolution. Integr Comp Biol. 56:644–56. 27252214 10.1093/icb/icw034 PMC6080509

[bib87] Seney ML , Kelly DA, Goldman BD, Sumbera R, Forger NG. 2009. Social structure predicts genital morphology in African mole-rats. PLoS ONE. 4:e7477. 19829697 10.1371/journal.pone.0007477 PMC2759003

[bib89] Sinclair AW , Glickman S, Catania K, Shinohara A, Baskin L, Cunha GR. 2017. Comparative morphology of the penis and clitoris in four species of moles (Talpidae). J Exp Zool B Mol Dev Evol. 328:275–94. 28251823 10.1002/jez.b.22732 PMC5448796

[bib88] Sinclair AW , Glickman SE, Baskin L, Cunha GR. 2016. Anatomy of mole external genitalia: setting the record straight. Anat Rec. 299:385–99. 10.1002/ar.23309 PMC4752857 26694958

[bib90] Spani F , Morigi MP, Bettuzzi M, Scalici M, Gentile G, Carosi M. 2021. The ultimate database to (re)set the evolutionary history of primate genital bones. Sci Rep. 11:11245. 34045627 10.1038/s41598-021-90787-2 PMC8160331

[bib91] Starck D. 1975. Embryologie. 3rd ed. Stuttgart: Georg Thieme Verlag Stuttgart. p. 519–20.

[bib92] Tripp HRH. 1971. Reproduction in elephant-shrews (Macroscelididae) with special reference to ovulation and implantation. Reproduction. 26:149–59. 10.1530/jrf.0.0260149 5558403

[bib93] Ulfelder H , Robboy SJ. 1976. The embryologic development of the human vagina. Am J Obstet Gynecol. 126:769–76. 1033667 10.1016/0002-9378(76)90666-9

[bib94] Van der Horst CJ. 1942. Some observations of the structure of reproductive tract of *Elephantulus*. J Morphol. 70:403.

[bib95] van Turnhout AA , Hage JJ, van Diest PJ. 1995. The female corpus spongiosum revisited. Acta Obstet Gynecol Scand. 74:767–71. 8533557 10.3109/00016349509021194

[bib96] Wagner GP. 2014. Homology, genes, and evolutionary innovation. Princeton (NJ): Princeton University Press.

[bib97] Watson M. 1879. The homology of the sexual organs illustrated by comparative anatomy and pathology. J Anat Physiol. 14:50–80. PMC1309916 17231308

[bib98] Watson M. 1881. On the female organs and placentation of the Racoon (*Procyon lotor*). Proc R Soc Lond. 32:272–98.

[bib99] Watson PF , Glover TE. 1993. Vaginal anatomy of the domestic cat (*Felis catus*) in relation to copulation and artificial insemination. J Reprod Fertil Suppl. 47:355–9. 8229949

[bib100] Weiss DA , Rodriguez E Jr., Cunha T, Menshenina J, Barcellos D, Chan LY, Risbridger G, Baskin L, Cunha G. 2012. Morphology of the external genitalia of the adult male and female mice as an endpoint of sex differentiation. Mol Cell Endocrinol. 354:94–102. 21893161 10.1016/j.mce.2011.08.009 PMC3717118

[bib101] Wells M. 1968. A comparison of the reproductive tracts of *Crocuta crocuta, Hyena hyena* and *Proteles cristatus*. Afr J Ecol. 6:63–70.

[bib102] Whitworth DJ , Licht P, Racey PA, Glickman SE. 1999. Testis-like steroidogenesis in the ovotestis of the European mole, *Talpa europaea*. Biol Reprod. 60:413–8. 9916009 10.1095/biolreprod60.2.413

[bib103] Wiedersheim R. 1908. Vergleichende Anatomie der Wirbeltiere. Jena: Gustav Fischer Verlag.

[bib104] Wislocki GB. 1936. The external genitalia of the simian primates. Hum Biol Rec Res. 8:309–47.

[bib105] Yamada G , Suzuki K, Haraguchi R, Miyagawa S, Satoh Y, Kamimura M, Nakagata N, Kataoka H, Kuroiwa A, Chen Y. 2006. Molecular genetic cascades for external genitalia formation: an emerging organogenesis program. Dev Dyn. 235:1738–52. 16598715 10.1002/dvdy.20807

