# Supplementary material for: Female Genital Variation Far Exceeds That of Male Genitalia: A Review of Comparative Anatomy of Clitoris and the Female Lower Reproductive Tract in Theria
Source: Integr Comp Biol. 2022 May 7;62(3):581–601. doi: 10.1093/icb/icac026 (PMC9494530; doi:10.1093/icb/icac026)
Supplement: icac026_Supplemental_File [file icac026_supplemental_file.docx]

**SUPPLEMENTARY MATERIAL**

**Table S1**: Coded anatomical data.

**Code** UGS (0: absent, 1: tubular, 2: flat vestibulum); vaginal segment (0: absent, 1: one, 2: two); corpus position (1: edge of long UGS, 2: in vestibulum, 4: in long UGS,5: outside); corpus perforation (0: no perforation, 1: perforated); glans lobes (1:none, 2: two lobes, 3: bipartite); cervix (0: absent, 1: present)

| **Species** | **UGS** | **Vaginal segment** | **Corpus c. position** | **Corpus c. perforation** | **Glans lobes** | **Cervix** |
| --- | --- | --- | --- | --- | --- | --- |
| *Sarcophilus harrisii* | 1 | 2 | 1 | 0 | 3 | 1 |
| *Monodelphis domestica* | 1 | 1 | 1 | 0 | 2 | 0 |
| *Didelphis virginiana* | NA | NA | 1 | 0 | 2 | NA |
| *Orycteropus afer* | 1 | 1 | NA | 0 | 2 | 1 |
| *Elephantulus myurus* | 0 | 1 | 0 | NA | NA | NA |
| *Trichechus manatus* | 1 | 1 | 1 | 0 | NA | 1 |
| *Procavia capensis* | 1 | 1 | NA | 0 | 1 | 1 |
| *Loxodonta africana* | 1 | 1 | 4 | 0 | 1 | 1 |
| *Elephas maximus* | 1 | 1 | 4 | 0 | 1 | 1 |
| *Bradypus variegatus* | 1 | 0 | NA | 0 | 2 | 0 |
| *Bradypus tridactylus* | 1 | 0 | 1 | 0 | 1 | 0 |
| *Tamandua tetradactyla* | 1 | 0 | NA | 0 | 1 | 0 |
| *Cyclopes didactylus* | NA | NA | NA | 0 | 2 | NA |
| *Dasypus novemcinctus* | 1 | 0 | 1 | 0 | 1 | 0 |
| *Tolypeutes matacus* | 1 | 1 | 2 | 0 | 1 | NA |
| *Chlamyphorus truncatus* | 1 | 0 | 1 | 0 | 1 | NA |
| *Euphractus sexcinctus* | 1 | 0 | 1 | 0 | 1 | NA |
| *Zaedyus pichiy* | 1 | 0 | 1 | 0 | 1 | NA |
| *Talpa europaea* | 0 | 1 | 5 | 0 | 1 | 1 |
| *Scapanus latimanus* | 0 | 1 | 5 | 0 | 1 | 1 |
| *Scalopus aquaticus* | 0 | 1 | 5 | 0 | 1 | 1 |
| *Felis catus* | 1 | 1 | 2 | 0 | 1 | 1 |
| *Crocuta crocuta* | 1 | 1 | 5 | 1 | 1 | 1 |
| *Procyon lotor* | 1 | 1 | NA | 0 | 1 | 1 |
| *Canis lupus* | 2 | 1 | 2 | 0 | 1 | 1 |
| *Equus caballus* | 1 | 1 | 4 | 0 | 1 | 1 |
| *Sus scrofa* | 1 | 1 | 4 | 0 | 1 | 1 |
| *Bos taurus* | 2 | 1 | 2 | 0 | 1 | 1 |
| *Bison bonasus* | 2 | 1 | 2 | 0 | 1 | 1 |
| *Phocoena phocoena* | 2 | 1 | 1 | 0 | 1 | 1 |
| *Balaena mysticetus* | 1 | 1 | NA | 0 | NA | 1 |
| *Sylvilagus floridanus* | 1 | 1 | 1 | 0 | 1 | 1 |
| *Oryctolagus cuniculus* | 1 | 1 | 1 | 0 | 1 | 1 |
| *Castor fiber* | 2 | 1 | 4 | 0 | 1 | 1 |
| *Mesocricetus auratus* | 0 | 1 | 5 | 0 | 1 | 1 |
| *Mus musculus* | 0 | 1 | 5 | 0 | 1 | 1 |
| *Rattus rattus* | 0 | 1 | 5 | 0 | 1 | 1 |
| *Spermophilus citellus* | 1 | 1 | 1 | 0 | 1 | 1 |
| *Spermophilus suslicus* | 1 | 1 | 1 | 0 | 1 | 1 |
| *Heterocephalus glaber* | 0 | 1 | 5 | 0 | 1 | 1 |
| *Fukomys damarensis* | 0 | 1 | 5 | 0 | 1 | 1 |
| *Heliophobius argenteocinereus* | 0 | 1 | 5 | 0 | 1 | 1 |
| *Myocastor coypus* | 0 | 1 | 2 | 0 | 1 | 1 |
| *Chinchilla chinchilla* | 1 | 1 | 5 | 0 | 1 | 1 |
| *Dasyprocta leporina* | 0 | 1 | 5 | 1 | 1 | 1 |
| *Cavia porcellus* | 2 | 1 | 5 | 0 | 1 | 1 |
| *Galea spixii* | 0 | 1 | 5 | 1 | 1 | 1 |
| *Tupaia splendidula* | 1 | 1 | 2 | 0 | 1 | 1 |
| *Lemur catta* | 0 | 1 | 2 | 1 | 1 | 1 |
| *Macaca mulatta* | 1 | 1 | 2 | 0 | 1 | 1 |
| *Hylobates lar* | 1 | 1 | 2 | 0 | 1 | 1 |
| *Hylobates agilis* | 1 | 1 | 2 | 0 | 1 | 1 |
| *Homo sapiens* | 2 | 1 | 2 | 0 | 1 | 1 |
| *Pan troglodytes* | 2 | 1 | 2 | 0 | 1 | 1 |
| *Pongo pygmaeus* | 2 | 1 | 2 | 0 | 1 | 1 |
| *Ateles geoffroyi* | NA | 1 | 2 | 0 | 1 | 1 |
| *Alouatta palliata* | 1 | 1 | 2 | 0 | 1 | 1 |
| *Leontopithecus chrysomelas* | NA | 1 | 2 | 0 | 1 | 1 |
| *Callimico goeldii* | 2 | 1 | 2 | 0 | 2 | 1 |
| *Callithrix geoffroyi* | 1 | 1 | 2 | 0 | 1 | 1 |
| *Aotus zonalis* | NA | 1 | 2 | 0 | 1 | 1 |
| *Cebus capucinus* | 1 | 1 | 2 | 0 | 1 | 1 |
| *Sapajus apella* | 2 | 1 | 2 | 0 | 1 | 1 |
| *Saimiri oerstedii* | NA | 1 | 2 | 0 | 1 | 1 |
| *Saimiri sciureus* | NA | 1 | 2 | 0 | 1 | 1 |
| *Saimiri vanzolinii* | NA | 1 | 2 | 0 | 1 | 1 |
